# Supplementary material for: Invasive Streptococcus agalactiae infections in infants in Guangzhou, Southern China (2013–2022): molecular epidemiology and clinical management implications
Source: BMC Microbiol. 2026 May 25;26:654. doi: 10.1186/s12866-026-05195-1 (PMC13386615; doi:10.1186/s12866-026-05195-1)
Supplement: Supplementary file 1 — Supplementary material 1. [file 12866_2026_5195_MOESM1_ESM.docx]

**Appendix Table 1: Primer sequences for GBS capsular serotype typing ^[11]^**

| **Primer name** | **Primer sequences (5'-3')** |
| --- | --- |
| cpsl-la-6-7-F | GAATTGATAACTTTTGTGGATTGCGATGA |
| cpsl-6-R | CAATTCTGTCGGACTATCCTGATG |
| cpsl-7-R | TGTCGCTTCCACACTGAGTGTTGA |
| cpsL-F | CAATCCTAAGTATTTTCGGTTCATT |
| cpsL-R | TAGGAACATGTTCATTAACATAGC |
| cpsG-F | ACATGAACAGCAGTTCAACCGT |
| CpsG-R | ATGCTCTCCAAACTGTTCTTGT |
| CpsG-2-3-6-R | TCCATCTACATCTTCAATCCAAGC |
| CpsN-5-F | ATGCAACCAAGTGATTATCATGTA |
| CpsN-5-R | CTCTTCACTCTTTAGTGTAGGTAT |
| CpsJ-8-F | TATTTGGGAGGTAATCAAGAGACA |
| CpsJ-8-R | GTTTGGAGCATTCAAGATAACTCT |
| cpsJ-2-4-F | CATTTATTGATTCAGACGATTACATTGA |
| cpsJ-2-R | CCTCTTTCTCTAAAATATTCCAACC |
| cpsJ-4-R | CCTCAGGATATTTACGAATTCTGTA |
| cpsl-7-9-F | CTGTAATTGGAGGAATGTGGATCG |
| cpsl-9-R | AATCATCTTCATAATTTATCTCCCATT |
| cpsJ-Ib-F | GCAATTCTTAACAGAATATTCAGTTG |
| cpsJ-Ib-R | GCGTTTCTTTATCACATACTCTTG |

Note: The concentrations of the primers cpsl-la-6-7-F and cpsl-7-9-F are 0.2 μl, whereas the concentrations of the remaining primers are 0.1 μ

**Appendix Table 2:The fragment lengths corresponding to each capsule serotype respectively^[11]^**

| **Ⅰa** | **Ⅰb** | **Ⅱ** | **Ⅲ** | **Ⅳ** | **Ⅴ** | **Ⅵ** | **Ⅶ** | **Ⅷ** | **Ⅸ** |
| --- | --- | --- | --- | --- | --- | --- | --- | --- | --- |
| 688bp  (cpsL) | 688bp  (cpsL) | 688bp  (cpsL) | 688bp  (cpsL) | 688bp  (cpsL) | 688bp  (cpsL) | 688bp  (cpsL) | 688bp  (cpsL) | 688bp  (cpsL) | 688bp  (cpsL) |
|  | 621bp  (cpsJ) |  |  |  |  |  |  |  |  |
|  |  |  |  |  | 582bp  (cpsN) |  |  |  |  |
|  |  |  |  | 538bp  (cpsJ) |  |  |  |  |  |
|  |  |  |  |  |  | 470bp  (cpsI) |  |  |  |
|  |  | 465bp  (cpsJ) |  |  |  |  |  |  |  |
|  |  |  |  |  |  |  |  | 438bp  (cpsJ) |  |
|  |  | 352bp  (cpsG) |  |  |  | 352bp  (cpsG) |  |  |  |
| 272bp  (cpsG) | 272bp  (cpsG) |  | 272bp  (cpsG) | 272bp  (cpsG) | 272bp  (cpsG) |  | 272bp  (cpsG) |  | 272bp  (cpsG) |
|  |  |  |  |  |  |  |  |  | 229bp  (cpsI) |
|  |  |  |  |  |  |  | 179bp  (cpsI) |  |  |

Note: The names in parentheses indicate the GBS capsule genes used to design the primers.

**Appendix Table 3: Sequence of GBS master gene primers and product sizes**

| **Primer name** | **Forward primer（5'→3'）** | **Reverse primer（5'→3'）** | **Product(bp)** |
| --- | --- | --- | --- |
| adhP | GTTGGTCATGGTGAAGCACT | ACTGTACCTCCAGCACGAAC | 672 |
| pheS | GATTAAGGAGTAGTGGCACG | TTGAGATCGCCCATTGAAAT | 723 |
| atr | CGATTCTCTCAGCTTTGTTA | AAGAAATCTCTTGTGCGGAT | 627 |
| glnA | CCGGCTACAGATGAACAATT | CTGATAATTGCCATTCCACG | 589 |
| sdhA | AGAGCAAGCTAATAGCCAAC | ATATCAGCAGCAACAAGTGC | 646 |
| glcK | CTCGGAGGAACGACCATTAA | CTTGTAACAGTATCACCGTT | 607 |
| tkt | CCAGGCTTTGATTTAGTTGA | AATAGCTTGTTGGCTTGAAA | 859 |

**Appendix Table 4: Primer sequences of Alp surface protein antigen gene, hylB, cylE virulence genes and Pilus islands^[20]^**

| **Genes** | **Primer name** | **Primer sequences** (5'-3') |
| --- | --- | --- |
| alpha and alpha-like surface protein genes | alp Universal-F | TGATACTTCACAGACGAAACAACG |
|  | alphaC-R | TACATGTGGTAGTCCATCTTCACC |
|  | rib-R | CATACTGAGCTTTTAAATCAGGTGA |
|  | alp1-R | CCAGATACATTTTTTACTAAAGCGG |
|  | alp2/3-R | CACTCGGATTACTATAATATTTAGCAC |
|  | alp4-R | TTAATTTGCACCGGATTAACACCAC |
| virulence genes | hylB | F: TCCATTTAAAGCCCTTGGTG |
|  |  | R: GGCGCCAGTATAAGCAACAT |
|  | cylE | F: TGACATTTACAAGTGACGAAG |
|  |  | R: TTGCCAGGAGGAGAATAGGA |
| Pilus islands | PI-1 | F: CTACCAACGGCCAAGCTATTTACC |
|  |  | R: TAGCCGCTTTTTCATTCTTTCTCC |
|  | PI-2a | F: AACTCCCTATATTTGCAGGTTCAA |
|  |  | R: CGGGTGTAACGACTTTTATCTGAT |
|  | PI-2b | F: GGGGGTAGGCTTAATGGCTTAT |
|  |  | R: TCCGGTTTAACTGTTCTGATTTGAT |
