# Supplementary material for: Invasive Streptococcus agalactiae infections in infants in Guangzhou, Southern China (2013–2022): molecular epidemiology and clinical management implications
Source: BMC Microbiol. 2026 May 25;26:654. doi: 10.1186/s12866-026-05195-1 (PMC13386615; doi:10.1186/s12866-026-05195-1)
Supplement: Supplementary file 3 — Supplementary material 3. [file 12866_2026_5195_MOESM3_ESM.docx]

1. Serotype electrophoresis results (partial)





1. Electrophoretic results of pilus islands genes


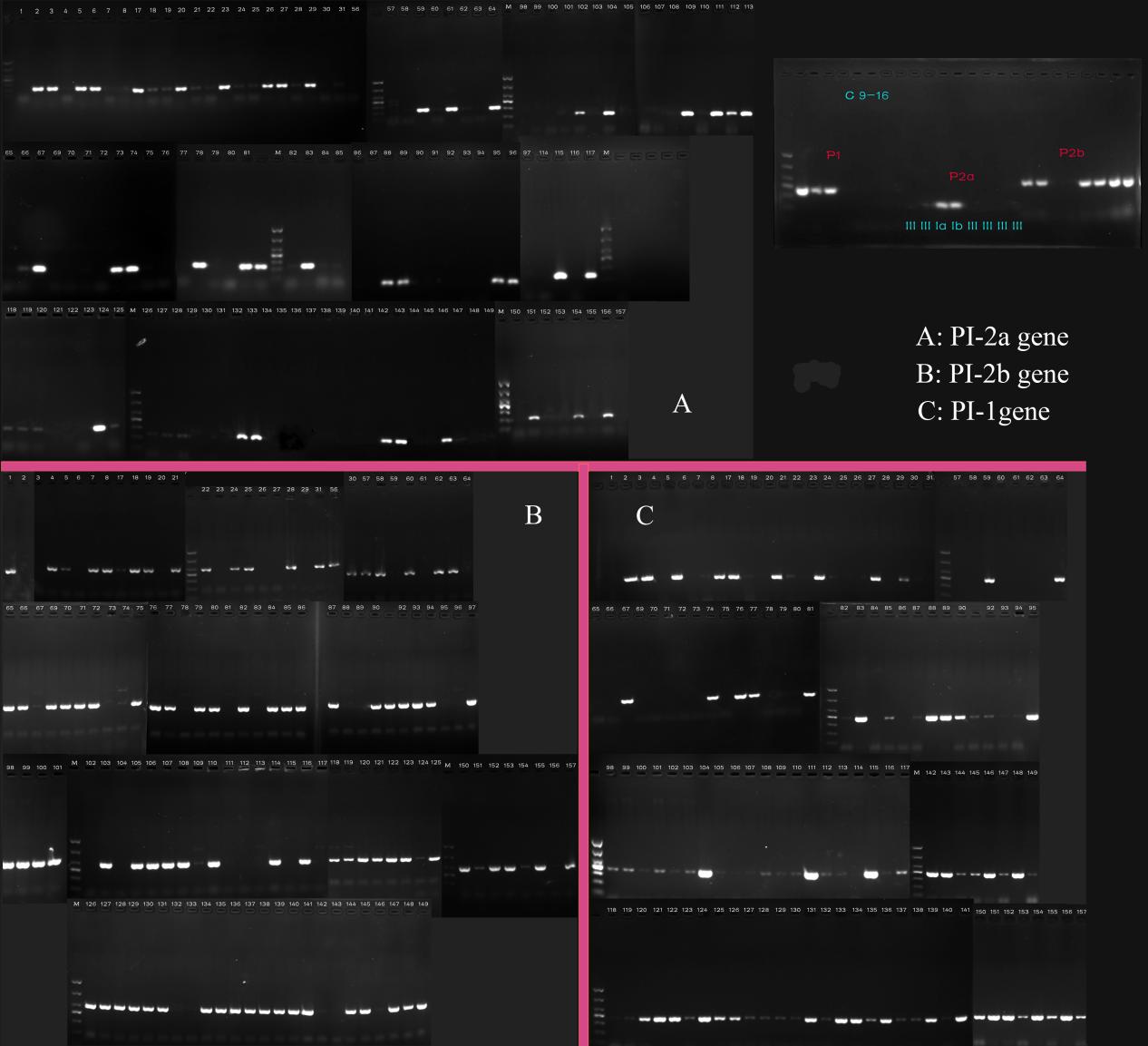


1.
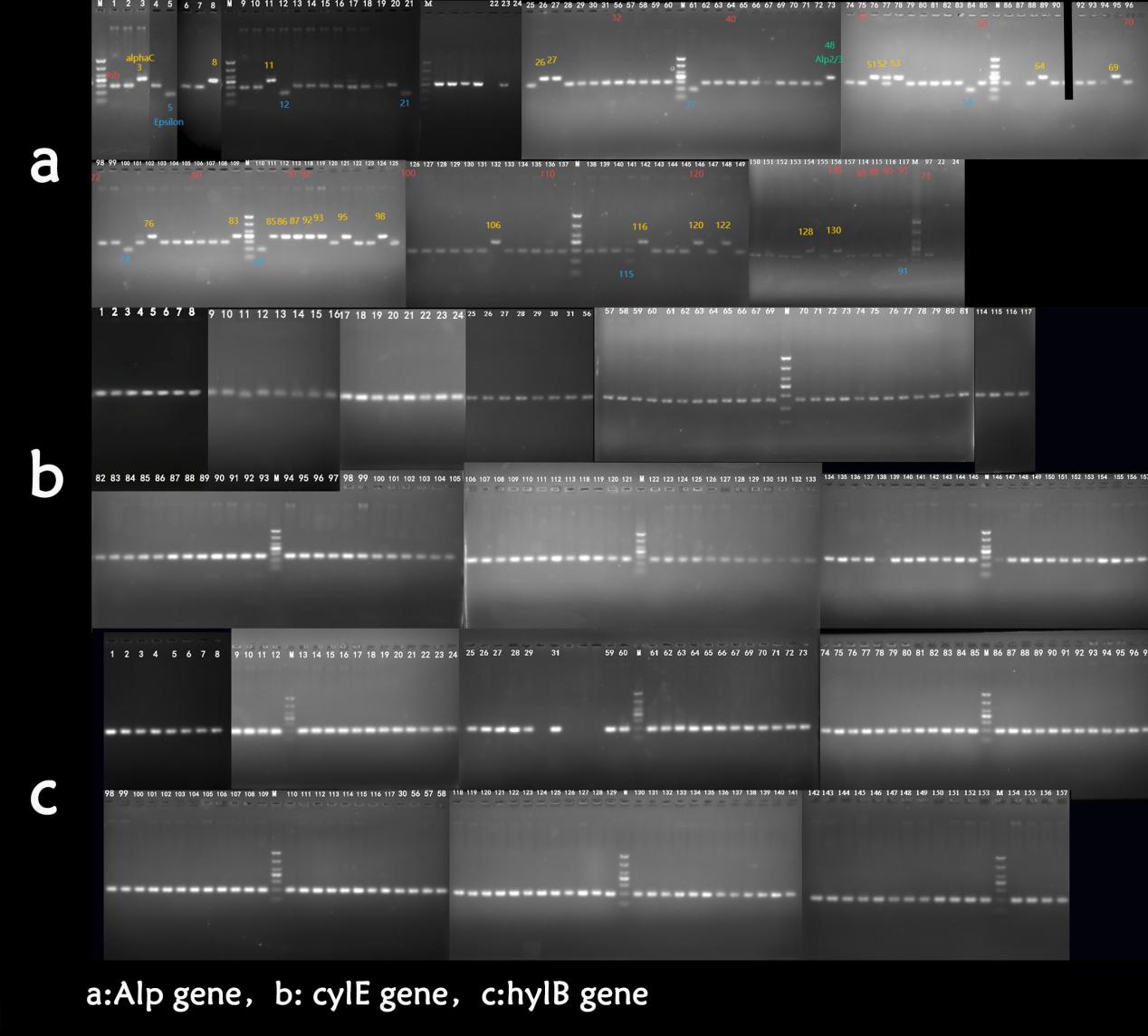
Electrophoretic results of ALP protein family and virulence genes of cylE and hylB
