# Supplementary figures and images for: Invasive Streptococcus agalactiae infections in infants in Guangzhou, Southern China (2013–2022): molecular epidemiology and clinical management implications
Source: BMC Microbiol. 2026 May 25;26:654. doi: 10.1186/s12866-026-05195-1 (PMC13386615; doi:10.1186/s12866-026-05195-1)

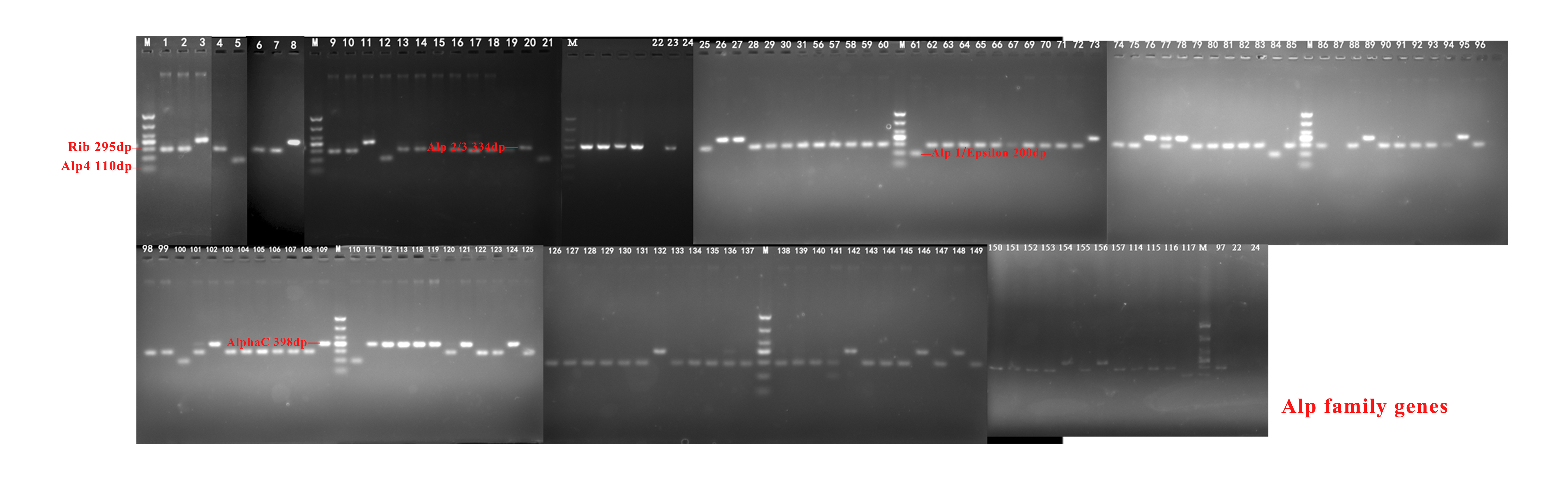

Supplement: Supplementary file 4 — Supplementary material 4. [file 12866_2026_5195_MOESM4_ESM.zip › electrophoresis results (supplement)/Alp family genes.jpg]

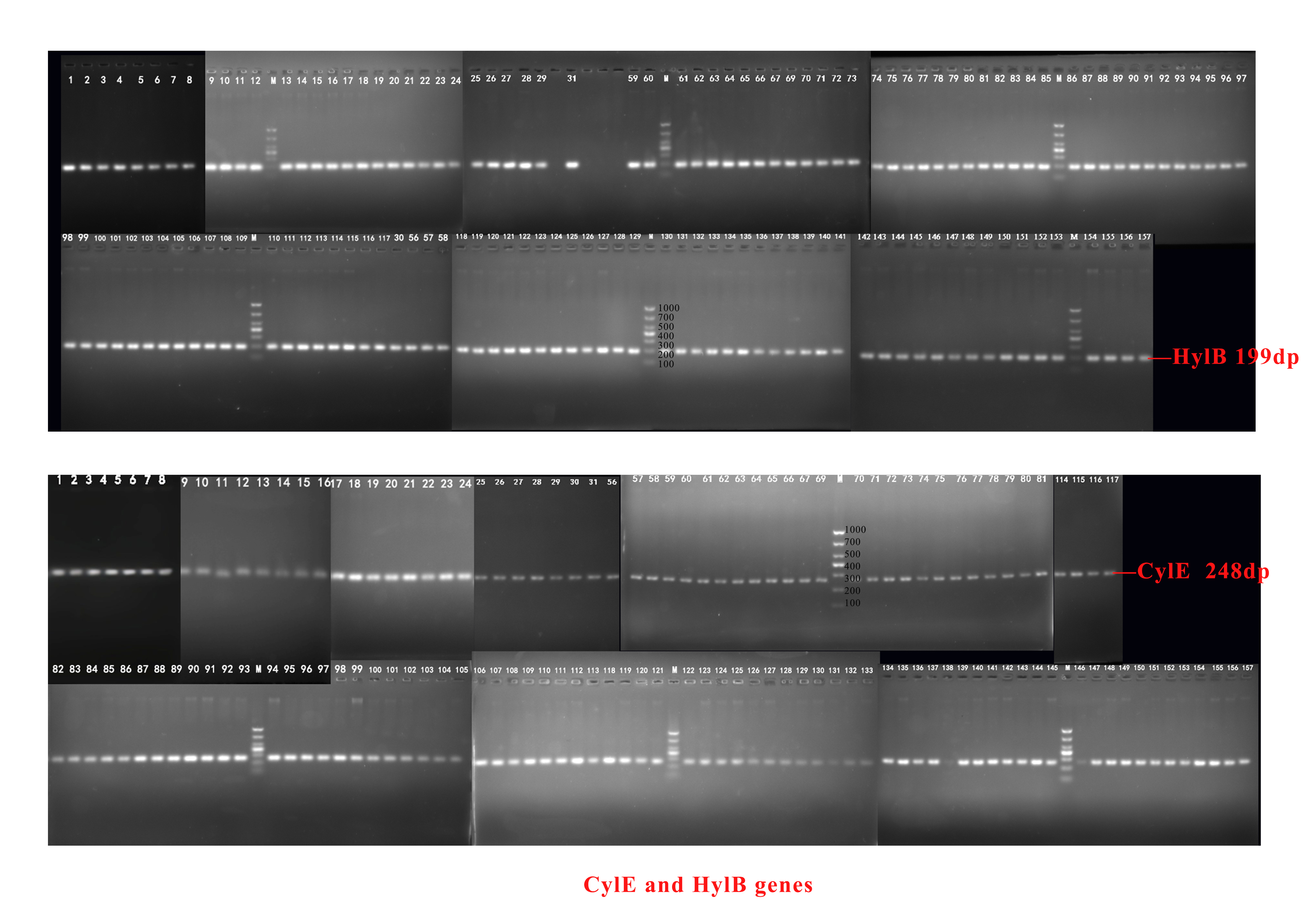

Supplement: Supplementary file 4 — Supplementary material 4. [file 12866_2026_5195_MOESM4_ESM.zip › electrophoresis results (supplement)/CylE and HylB genes.jpg]

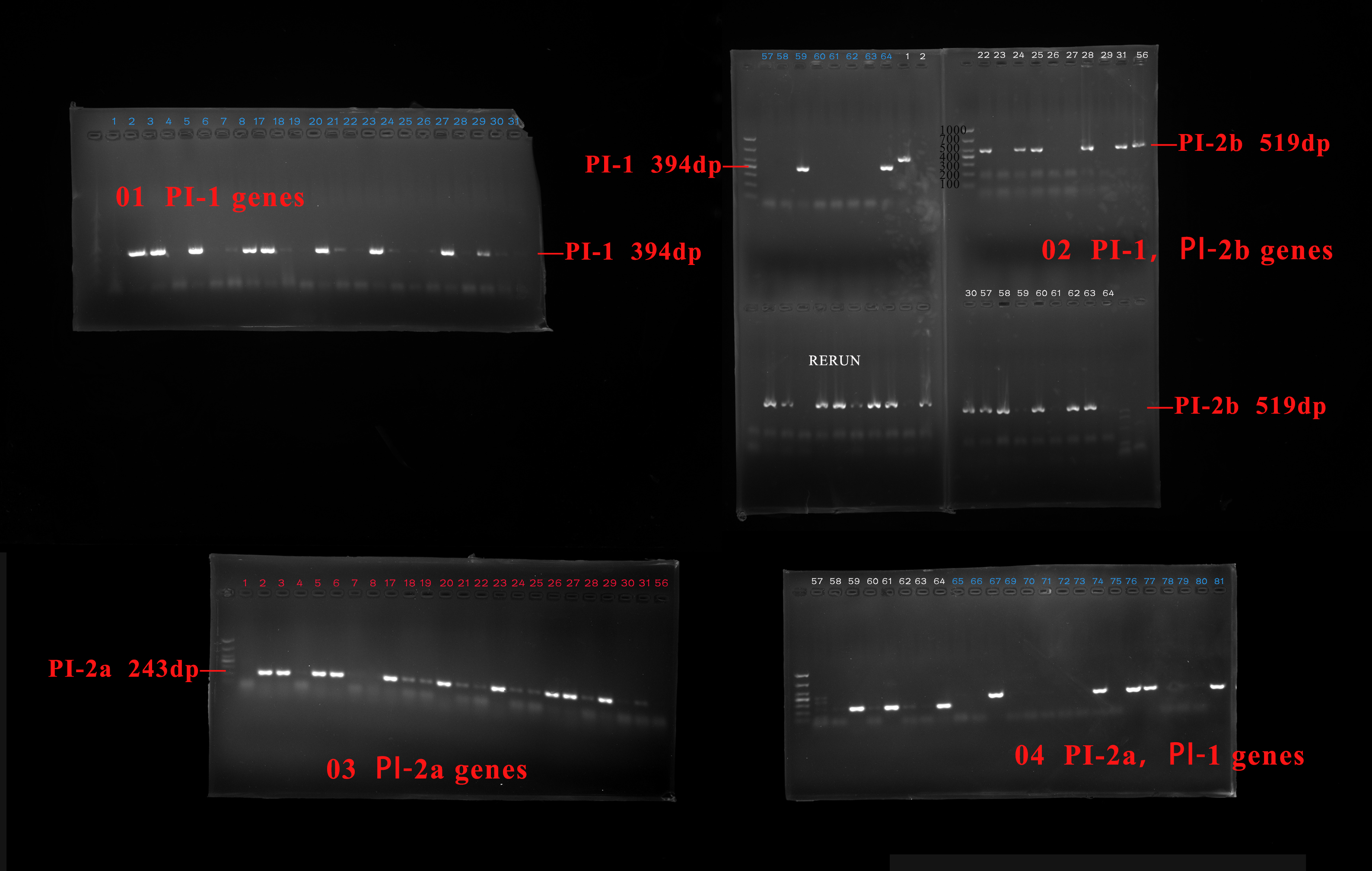

Supplement: Supplementary file 4 — Supplementary material 4. [file 12866_2026_5195_MOESM4_ESM.zip › electrophoresis results (supplement)/pilus island 01-04.jpg]

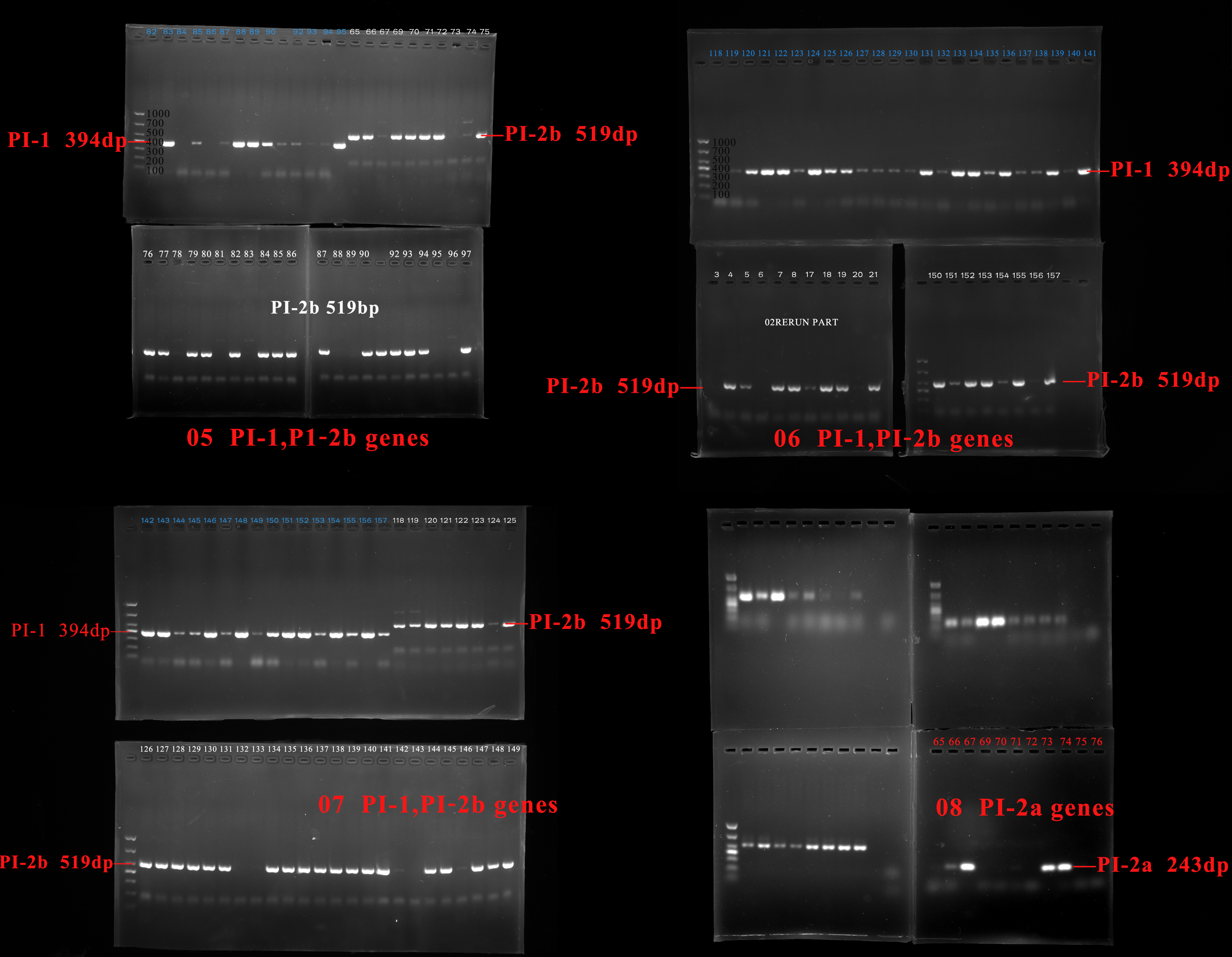

Supplement: Supplementary file 4 — Supplementary material 4. [file 12866_2026_5195_MOESM4_ESM.zip › electrophoresis results (supplement)/pilus islands 05-08.jpg]

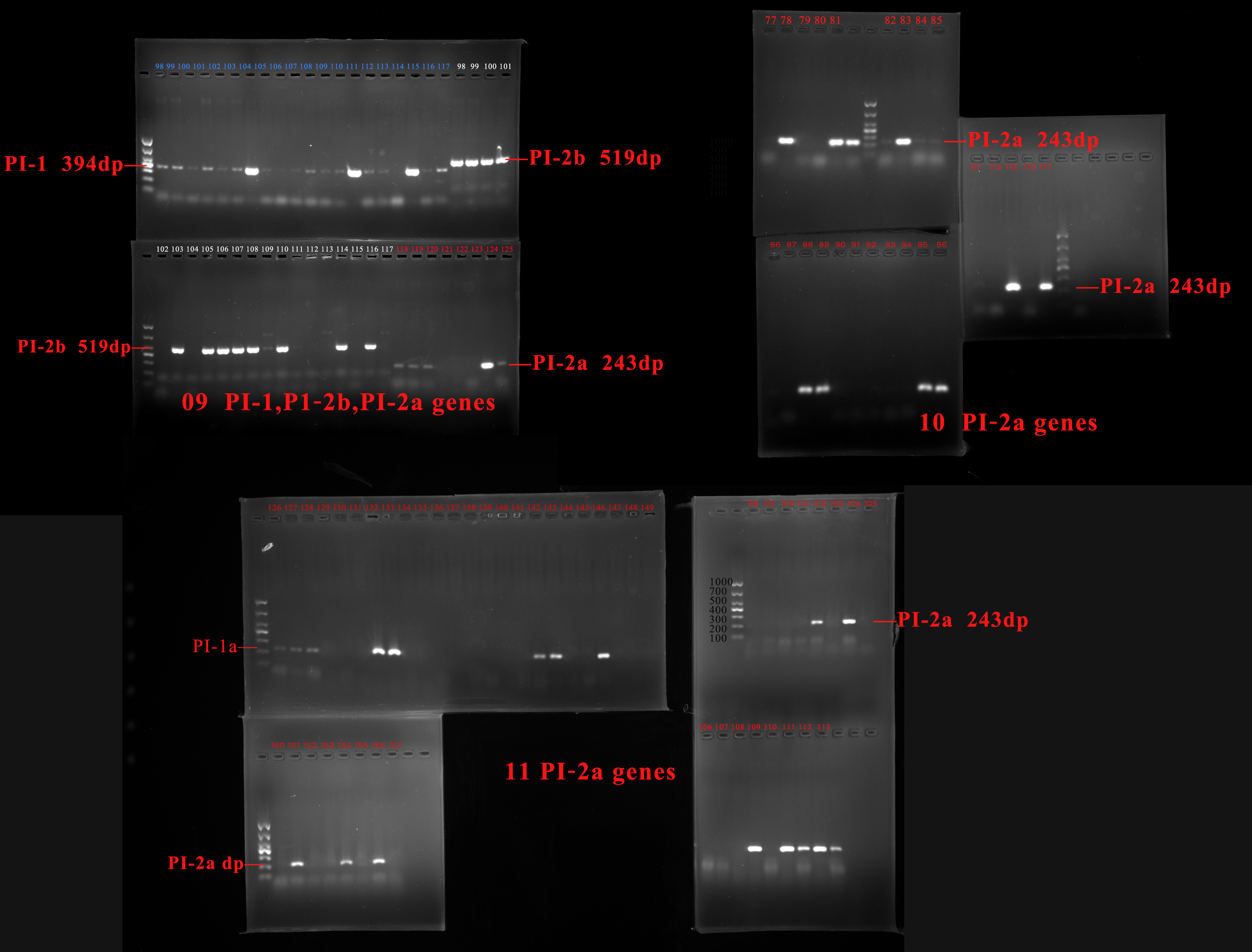

Supplement: Supplementary file 4 — Supplementary material 4. [file 12866_2026_5195_MOESM4_ESM.zip › electrophoresis results (supplement)/pilus islands 09-11.jpg]

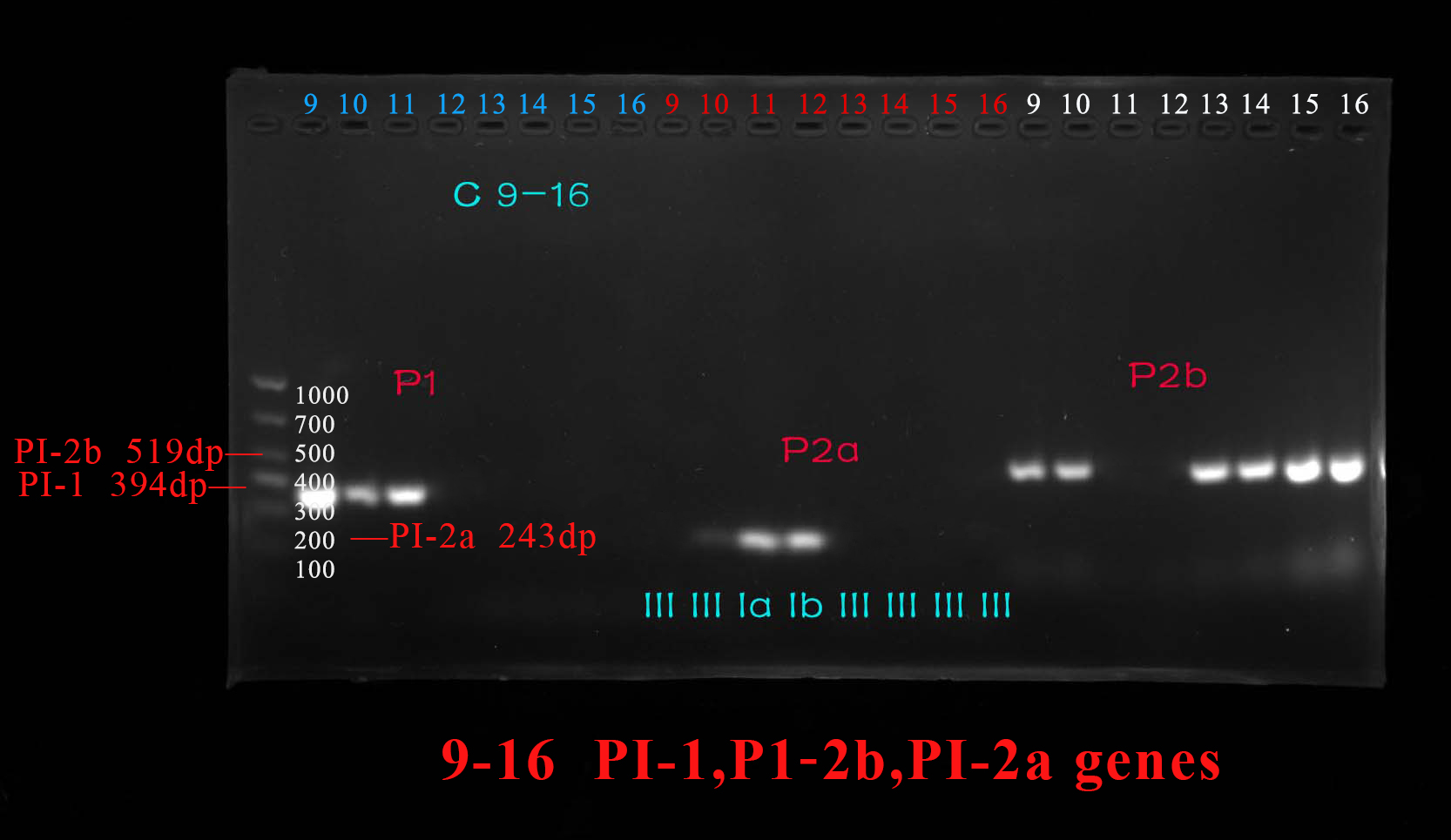

Supplement: Supplementary file 4 — Supplementary material 4. [file 12866_2026_5195_MOESM4_ESM.zip › electrophoresis results (supplement)/pilus islands 12.jpg]

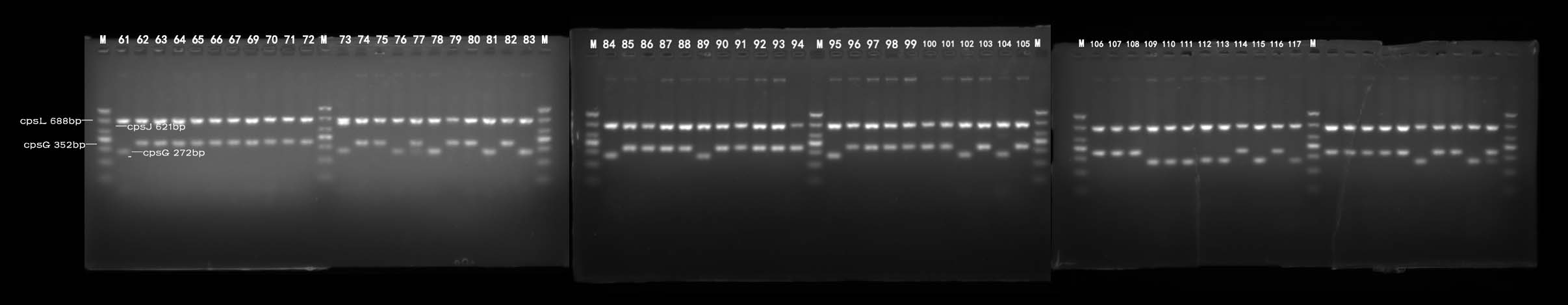

Supplement: Supplementary file 4 — Supplementary material 4. [file 12866_2026_5195_MOESM4_ESM.zip › electrophoresis results (supplement)/Serotype electrophoresis results (partial).jpg]
