# Supplementary material for: Invasive Streptococcus agalactiae infections in infants in Guangzhou, Southern China (2013–2022): molecular epidemiology and clinical management implications
Source: BMC Microbiol. 2026 May 25;26:654. doi: 10.1186/s12866-026-05195-1 (PMC13386615; doi:10.1186/s12866-026-05195-1)
Supplement: Supplementary file 5 — Supplementary material 5. [file 12866_2026_5195_MOESM5_ESM.pdf]

广州医科大学附属妇女儿童医疗中心科研伦理委员会

伦理审查批件

穗妇儿 科伦 批字[2025]第 120A01 号

**项目名称**	孕妇和低龄婴儿无乳链球菌感染性疾病临床特征及分离菌分子特征研究		
**负责人**	黄莲芬	**专业部门/科室**	临床检验部/儿童院区检验科
**伦理联系人**	黄莲芬	**联系方式**	18198915226
**研究期限**	2015.7.1-2024.12.31	**审查类别**	**快速审查**
**审查文件**	1.涉及人的生物医学实验伦理审查申请表<br>2.研究者履历<br>3.研究方案		
**审查结果**	根据中华人民共和国国家卫计委第 11 号令《涉及人的生物医学研究伦理审查办法》以及《赫尔辛基宣言》和国际医学科学组织委员会颁布的《人体生物医学研究国际道德指南》等伦理原则，本伦理委员会审查决定为：**同意。**		
**主审委员**	黄海、李杰		
**意见说明**	在研究进行过程中研究者请按照伦理委员会规定的跟踪审查频率，申请人在截止日期前 1 个月提交研究进展报告。研究负责人必须严格使用经审查同意的知情同意书文本和研究方案。如伦理审查批件失效时不能完成所有的临床研究（包括统计分析），请在本批件失效前一个月，递交持续审查申请。如研究结束并在审查有效期内，请递交研究结题报告。研究中发生涉及受试者或其他人风险的任何 SAE 或非预期的不良事件，应立刻报告本伦理委员会；任何研究方案、知情同意书的修改包括研究人员的变更，必须递交修正案审查申请；若提前终止或暂停研究，请及时提交暂停/终止研究报告。出现违背方案或可能对受试者权益/健康及研究的科学性造成不良影响等违背科研伦理原则的情况，应及时提交违背方案报告。		
**批件有效期**	2015.7.1-2024.12.31	**跟踪审查频率**	12 个月
**主任（副主任）委员签名**		**日期**	2025 年 [illegible] 13 日
**伦理委员会**	广州医科大学附属妇女儿童医疗中心科研伦理委员会（盖章）		

广州医科大学附属妇女儿童医疗中心科研伦理委员会

联系电话：020-38367270
