# Supplementary material for: Invasive Streptococcus agalactiae infections in infants in Guangzhou, Southern China (2013–2022): molecular epidemiology and clinical management implications
Source: BMC Microbiol. 2026 May 25;26:654. doi: 10.1186/s12866-026-05195-1 (PMC13386615; doi:10.1186/s12866-026-05195-1)
Supplement: Supplementary file 6 — Supplementary material 6. [file 12866_2026_5195_MOESM6_ESM.docx]

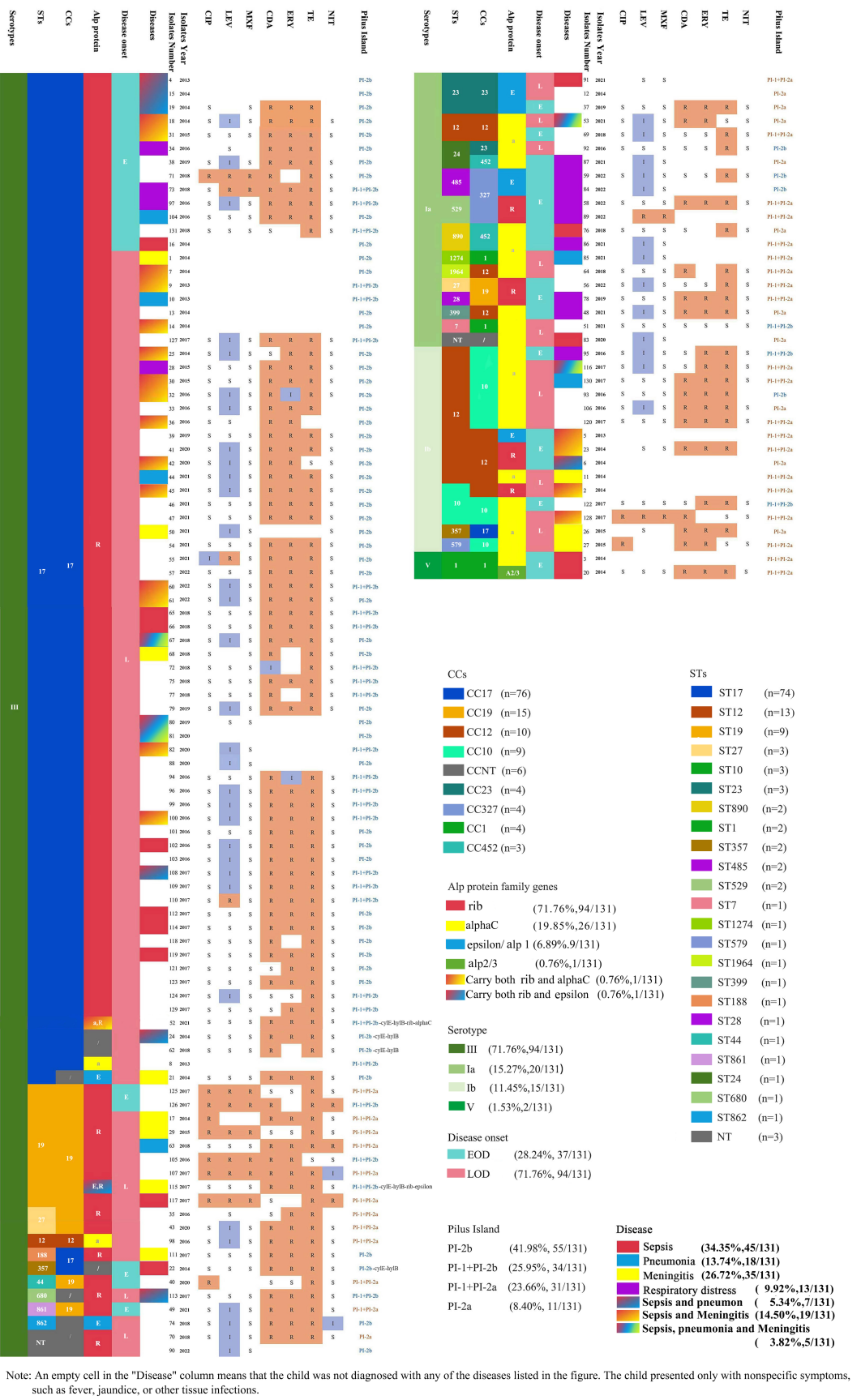


**Figure S1. Comprehensive molecular and clinical distribution of 131 invasive *Streptococcus agalactiae* (GBS) isolates**

Each column from left to right shows: serotype, sequence type (ST), clonal complex (CC), ALP family protein genes, disease onset type, clinical diagnosis, isolate count, collection year, antimicrobial susceptibilities, and pilus islands (PI) profiles. Different colors indicate distinct phenotypes within each category.

**Abbreviations**: GBS, group B *Streptococcus*; EOD, early-onset disease (0－6 days); LOD, late－onset disease (7-89 days); NT, not tested; R, resistant.

**Table S1. Annual antimicrobial resistance profiles of invasive GBS isolates, 2014－2022**

| **Year** | **Item** | **CIP** | **LEV** | **MXF** | **CLI** | **ERY** | **TET** | **NIT** |
| --- | --- | --- | --- | --- | --- | --- | --- | --- |
| **2014** | n | 8 | 7 | 8 | 9 | 9 | 9 | 7 |
|  | R (n,%) | 1 (12.5) | 0 | 0 | 8 (88.9) | 9 (100) | 9 (100) | 0 |
|  | I (n,%) | 0 | 2 (28.6) | 0 | 0 | 0 | 0 | 0 |
| **2015** | n | 5 | 5 | 4 | 6 | 6 | 6 | 5 |
|  | R (n,%) | 2 (40.0) | 1 (20.0) | 1(25.0) | 5 (83.3) | 5 (83.3) | 5 (83.3) | 0 |
|  | I (n,%) | 0 | 0 | 0 | 0 | 0 | 0 | 0 |
| **2016** | n | 18 | 20 | 17 | 20 | 20 | 19 | 15 |
|  | R (n,%) | 1 (5.6) | 1 (5.0) | 1 (5.9) | 17 (85.0) | 17 (85.0) | 18 (94.7) | 0 |
|  | I (n,%) | 0 | 11 (57.9) | 0 | 0 | 2 (18.2) | 0 | 0 |
| **2017** | n | 24 | 24 | 24 | 24 | 20 | 24 | 24 |
|  | R (n,%) | 5 (20.8) | 6 (25.0) | 5 (20.8) | 17 (70.8) | 18 (90.0) | 23 (95.8) | 2 (8.3) |
|  | I (n,%) | 0 | 5 (20.8) | 0 | 0 | 0 | 0 | 1 (4.2) |
| **2018** | n | 17 | 16 | 17 | 17 | 9 | 17 | 17 |
|  | R (n,%) | 1 (5.9) | 2 (12.5) | 2 (11.8) | 13 (76.5) | 8 (88.9) | 17 (100) | 1 (5.9) |
|  | I (n,%) | 0 | 4 (25.0) | 0 | 1 (5.9) | 0 | 0 | 1 (5.9) |
| **2019** | n | 5 | 6 | 6 | 5 | 5 | 5 | 5 |
|  | R (n,%) | 0 | 0 | 0 | 5 (100) | 5 (100) | 5 (100) | 0 |
|  | I (n,%) | 0 | 2 (33.3) | 0 | 0 | 0 | 0 | 0 |
| **2020** | n | 4 | 6 | 6 | 4 | 4 | 4 | 4 |
|  | R (n,%) | 1 (25.0) | 0 | 0 | 3 (75.0) | 3 (75.0) | 3 (75.0) | 0 |
|  | I (n,%) | 0 | 6 (100) | 0 | 0 | 0 | 0 | 0 |
| **2021** | n | 11 | 16 | 16 | 11 | 11 | 11 | 11 |
|  | R (n,%) | 0 | 1 (6.3) | 0 | 10 (90.9) | 10 (90.9) | 9 (81.2) | 0 |
|  | I (n,%) | 0 | 9 (56.3) | 0 | 0 | 0 | 0 | 0 |
| **2022** | n | 6 | 9 | 9 | 6 | 6 | 6 | 6 |
|  | R (n,%) | 0 | 1 (11.1) | 1 (11.1) | 4 (66.7) | 4 (66.7) | 6 (100) | 0 |
|  | I (n,%) | 0 | 6 (66.7) | 0 | 0 | 0 | 0 | 0 |
| **Overall** | n | 98 | 109 | 107 | 102 | 90 | 101 | 94 |
|  | R (n,%) | 11 (11.2) | 12 (11.0) | 10 (9.3) | 82 (80.4) | 79 (87.7) | 95 (94.1) | 3 (3.2) |
|  | I (n,%) | 0 | 45 (41.3) | 0 | 1 (1.0) | 2 (2.2) | 0 | 2 (2.1) |

**Abbreviations**: R, resistance; I, intermediate rate; n, number; CIP, ciprofloxacin; LEV, levofloxacin; MXF, moxifloxacin; CLD, clindamycin; ERY, erythromycin; TET, tetracycline; NIT, nitrofurantoin.

**Note**: Denominators vary owing to missing susceptibility data in early years and differing test panels.

**Table S2. Statistical associations between molecular features, antimicrobial resistance, and clinical phenotypes**

1. Associations between ST19 and quinolone resistance

| **Antibiotic** | **ST19（n=9）** | **Non-ST19** | ***P*-value** |
| --- | --- | --- | --- |
| CIP（n=98） | 7 | 4 | <0.001 |
| LEV（n=109） | 6 | 6 | <0.001 |
| MXF（n=107） | 6 | 4 | <0.001 |

1. Associations between clinical manisfestations, serotypes and disease onset

| **Feature** | **LOD(n=94)** | **EOD(n=37)** | ***P*-value** |
| --- | --- | --- | --- |
| Sepsis | 32 | 13 | 1.000 |
| Meningitis | 31 | 4 | 0.010 |
| Pneumonia | 13 | 5 | 1.000 |
| Respiratory distress | 1 | 12 | <0.001 |
| Serotype Ⅲ | 76 | 18 | <0.001 |
| Serotype Ⅰa | 8 | 12 | 0.001 |
| Serotype Ⅰb | 10 | 5 | 0.642 |
| Serotype Ⅴ | 0 | 2 | 0.078 |

1. Associations between serotype Ia and respiratory distress

| **Condition** | **Ia** | **non-Ia** | ***P*-value** |
| --- | --- | --- | --- |
| Respiratory distress | 8 | 5 | <0.001 |
| Non-respiratory distress | 12 | 106 |  |

1. Associations between rib gene, disease onset and III/ST17 clone

| **Feature** | | **III/ST17** | **non-III/ST17** | ***P*-value** | | |
| --- | --- | --- | --- | --- | --- | --- |
| rib | 70 | | 24 | | <0.001 |  |
| Non-rib | 4 | | 35 | |  |  |
| LOD | 61 | | 33 | | 0.002 |  |
| EOD | 13 | | 24 | |  |  |

**Note:***P* values were calculated using Fisher’s exact test or chi-square test.
