# Supplementary material for: Invasive Streptococcus agalactiae infections in infants in Guangzhou, Southern China (2013–2022): molecular epidemiology and clinical management implications
Source: BMC Microbiol. 2026 May 25;26:654. doi: 10.1186/s12866-026-05195-1 (PMC13386615; doi:10.1186/s12866-026-05195-1)
Supplement: Supplementary file 7 — Supplementary material 7. [file 12866_2026_5195_MOESM7_ESM.docx]

**Invasive *Streptococcus agalactiae* Infections in Infants in Guangzhou, Southern China (2013-2022): Molecular Epidemiology and Clinical Management Implications**

Xiao-lan Chen^1,#^, Hua-min Zhong^1,#^, Yong-qiang Xie^1,#^, Wen-shan Chen^1^, Li-Jia Wei^1^, Kan-kan Gao^1^, Bing-shao Liang^1,^ Su-fei Zhu^1^, Yun-feng Liu^1^, Fei Gao^1^, Yan Long^1,^*, Lian-fen Huang^1,^*

^1^Department of Laboratory Medicine, Guangzhou Medical University Affiliated Women and Children’s Medical Center, Guangdong Provincial Clinical Research Center for Laboratory Medicine, Guangzhou, China;

#These authors contributed equally to this work.

*Corresponding authors: hlfen2008@163.com (Lian-fen Huang); [Longyangmc@163.com](mailto:Longyangmc@163.com) (Yan Long)

**Abstract** *Streptococcus agalactiae* (group B *Streptococcus*, GBS) is an important cause of invasive infections in infants worldwide, despite widespread implementation of intrapartum antibiotic prophylaxis (IAP). This study aimed to clarify the molecular and clinical characteristics of invasive GBS isolates from infants in Guangzhou, China, over the 10-year period 2013－2022. A total of 131 GBS strains were recovered from blood cultures (n=98) and cerebrospinal fluid cultures (n=33) of infants aged ≤90 days. All isolates were subjected to capsular serotyping, multilocus sequence typing (MLST), virulence gene profiling (including *cylE*, *hylB*, Alp family genes, and pilus island genes), and antimicrobial susceptibility testing. Of the 131 GBS isolates, 37 (28.2%) caused early-onset disease (EOD) and 94 (71.8%) caused late-onset disease (LOD). The hypervirulent III/ST17 clone was the predominant lineage (56.5%) and was associated with LOD. Serotype Ia accounted for 15.3% of all isolates and was correlated with respiratory distress in EOD cases. All isolates carried the *cylE* and *hylB* toxin genes, with *rib* being the most prevalent surface protein gene (71.8%). Pilus island *PI-2b* (67.9%) was the dominant type and was predominantly detected in serotype III/CC17 strains. Resistance rates to clindamycin, erythromycin, and tetracycline exceeded 80%, while all isolates remained susceptible to penicillin and ampicillin. This study confirms that the III/ST17 clone is the predominant lineage associated with GBS LOD and identifies an association between serotype Ia and respiratory distress in GBS EOD. These findings support ongoing molecular surveillance to guide targeted preventive strategies and improve clinical management of invasive infantile GBS disease.

**Keywords:** *Streptococcus agalactiae*; Infants; ST17; Serotype Ia; Disease Management; Molecular Epidemiology

1. **Introduction**

*Streptococcus agalactiae* (group B *Streptococcus*, GBS) is a Gram-positive coccus and a common commensal organism colonizing the lower genital tract and rectum in women. It is also a major pathogen responsible for neonatal sepsis, meningitis, and pneumonia globally, often leading to severe outcomes, including neurological sequelae and neonatal death^1^. Clinically, invasive GBS infections in infants are classified into two distinct syndromes: early-onset disease (EOD), occurring at 0－6 days of life, and late-onset disease (LOD), occurring at 7－89 days of life. Multicenter clinical and molecular epidemiological analyses in China have reported an incidence of 0.31 cases of invasive GBS disease per 1000 live births, with a mortality rate of 2.3%^2^. Although intrapartum antibiotic prophylaxis (IAP) has been associated with a reduction in the incidence of EOD in many high-income countries, GBS continues to impose a substantial clinical burden, largely due to the limited efficacy of IAP for preventing LOD^3^.

GBS encodes a diverse array of virulence factors^4^, including pilus islands (PI), which play a pivotal role in biofilm formation^5^—a mechanism that may enable GBS to evade IAP. The Alp family of surface proteins mediates endothelial adhesion and represents a promising target for protein-based vaccines^6^. Meanwhile, the potent toxins hemolysin and hyaluronidase, encoded by the *cylE* and *hylB* genes, respectively, induce host cell damage and facilitate invasion across the placental barrier^7,8^. However, comprehensive analyses integrating the distribution of these key virulence factors in clinical GBS isolates remain limited.

Molecular characterization is useful for exploring the epidemiology and pathogenic characteristics of GBS. Traditional capsular serotyping based on capsular polysaccharide (CPS) antibodies^9^ remains widely used but has limitations for isolates with low or absent capsule expression. Geno-serotyping, combining latex agglutination with PCR-based capsular gene detection, has been developed to address this shortcoming and enable more accurate characterization^10,11^. Multilocus sequence typing (MLST) has been a standard method for defining GBS genetic lineages since 2003^12^. Accumulating evidence indicates that distinct GBS sequence types (STs) and clonal complexes (CCs) exhibit notable differences in pathogenic potential, epidemiological patterns, and antibiotic resistance profiles^13,14^. Our previous work demonstrated the dominance of the hypervirulent III/ST17 lineage in China, as well as its association with the meningeal tropism gene *hvgA* and major resistance genes^15^. Other studies have further linked specific STs with distinct resistance profiles: ST17 is frequently resistant to tetracycline but susceptible to levofloxacin, whereas ST19 tends to show the opposite pattern^16^; ST10 is associated with high radezolid minimum inhibitory concentrations (MICs) and fluoroquinolone resistance^17,18^; and the III/ST19 and Ib/ST10 lineages have been identified as major multidrug-resistant clones among fluoroquinolone-resistant GBS strains in China^19^.

Despite these advances, longitudinal epidemiological analyses of invasive GBS isolates from infants remain limited. In this study, we conducted a ten-year (2013–2022) retrospective molecular epidemiological investigation of invasive GBS isolates from infants aged ≤90 days in Guangzhou to characterize the distribution of serotypes, STs, and ten key virulence genes—including three pilus islands (*PI-1*, *PI-2a*, and *PI-2b*); five Alp family surface proteins (*rib*, *alphaC*, *alp1*, *alp2/3*, and *alp4*); and two toxin genes (*cylE* and *hylB*). This characterization may help inform more targeted GBS prevention strategies and improve the clinical management of invasive GBS disease in infants.

1. **Materials and Methods**

**2.1 Bacterial Isolates and Ethical Approval**

All GBS isolates were collected from blood cultures (n=98) and cerebrospinal fluid (CSF) cultures (n=33) of infants aged ≤90 days hospitalized at Guangzhou Medical University Affiliated Women and Children’s Medical Center during 2013-2022. Species identification was confirmed using matrix-assisted laser desorption/ionization time-of-flight mass spectrometry (MALDI-TOF MS). Relevant clinical data were retrieved from the hospital’s electronic medical records. The study protocol was approved by the Institutional Ethics Committee of the hospital (approval no. 2025120A01).

**2.2 Capsular Serotyping and MLST**

Molecular CPS serotyping was performed using a validated multiplex PCR assay and serum agglutination method^11^. Genomic DNA was extracted from GBS isolates using the SteadyPure Bacterial Genomic DNA Extraction Kit (Realy Biotechnology, China) following the manufacturer’s protocol for Gram-positive bacteria. MLST was performed by sequencing seven housekeeping genes (*adhP*, *pheS*, *atr*, *glnA*, *sdhA*, *glcK*, and *tkt*), and allele profiles, STs, and CCs were assigned by querying the PubMLST database (https://pubmlst.org/organisms/streptococcus-agalactiae/primers).

The goeBURST algorithm was applied to assign STs to CCs and to visualize and deduce the evolutionary genetic relationship and founder genotype among all recovered GBS isolates.

**2.3 Virulence Gene Detection**

PCR or multiplex PCR assays were used to detect the presence of three pilus island genes (*PI-1, PI-2a*, and *PI-2b*), five Alp family surface protein genes (*rib*, *alphaC*, *alp1*, *alp2/3*, and *alp4*), and two toxin genes (*cylE* and *hylB*)^20^.

**2.4 Antimicrobial Susceptibility Testing**

The MICs of 13 antibiotics (penicillin, ampicillin, ciprofloxacin, levofloxacin, moxifloxacin, linezolid, clindamycin, quinupristin/dalfopristin, erythromycin, vancomycin, tetracycline, nitrofurantoin, and tigecycline) were determined using the VITEK 2 Compact system (BioMérieux, Marcy-l’Étoile, France). Antimicrobial susceptibility interpretations were based on the 2020 Clinical and Laboratory Standards Institute (CLSI) criteria. The double-disk agar diffusion test (D-test) was used to detect the inducible macrolide–lincosamide–streptogramin B (iMLSB) phenotype. Multidrug resistance (MDR) was defined as resistance to at least three classes of antimicrobial drug agents.

**2.5 Statistical Analysis**

Categorical data were presented as numbers and percentages. Between-group comparisons of categorical variables were performed using the chi-square test or Fisher’s exact test (two-tailed). Binary logistic regression was performed to identify independent factors associated with the study periods (2018－2022 vs. 2013－2017). A *P value* ≤ 0.05 was considered statistically significant. All statistical analyses were conducted using SPSS software (version 26.0; IBM Corp., Armonk, NY, USA). A minimum spanning tree was generated using PHYLOVIZ version 2.0 to visualize genetic relationships between serotypes, STs, and CCs.

1. **Results**

**3.1 Epidemiology of Invasive GBS Infections**

Between 2013 and 2022, a total of 48,264 blood or CSF cultures were processed for hospitalized infants (≤90 days) at the study center, of which 137 (2.8%) were positive for GBS. Six isolates were excluded due to unsuccessful preservation, resulting in a final study cohort of 131 isolates. Clinical and demographic characteristics of infants with EOD and LOD are summarized in **Table 1** and **Figure 1**. Of the 131 isolates, 37 (28.2%) caused EOD and 94 (71.8%) caused LOD. The main clinical manifestations of EOD were septicemia (35.1%, 13/37) and respiratory distress (32.4%, 12/37), while LOD was predominantly characterized by septicemia (34.0%, 32/94) and meningitis (33.0%, 31/94). Other clinical manifestations included pneumonia (13.7%, 18/131), respiratory distress (9.9%, 13/131), pathological jaundice (7.6%, 10/131), and focal infections (bones, joints, soft tissues; 2.3%, 3/131). The mortality rate was higher in EOD patients (8.6%, 3/35, e**xclude missing values**) than in LOD patients (2.8%, 2/71, e**xclude missing values**). Notably, 80% of fatal cases (4/5, *P*<0.05) involved low birthweight infants (<2500 g).

Approximately 86.8% of pregnant women lacked GBS screening or had no documented results, and two-thirds of available screening records were obtained after August 2021. Prenatal GBS colonization status was documented for 14 mothers: the colonization rate was higher among mothers of infants with EOD (50.0%, 4/8) than among those of infants with LOD (33.3%, 2/6), although this difference was not statistically significant, likely due to the small sample size. Among GBS-colonized mothers, only one mother of an infant with EOD received IAP. One LOD case occurred in an infant born via in vitro fertilization, whose mother had a history of recurrent miscarriage (G8P3, recurrent pregnancy loss in G2 to G6). Overall, 8.4% (11/131) of mothers had a history of adverse pregnancy outcomes (e.g., spontaneous abortion or fetal demise). Additionally, one infant with LOD sepsis had an older sibling with a history of GBS meningitis. Among LOD cases with meningitis, three infants developed long-term sequelae following treatment, including focal epilepsy, speech delay, and autism spectrum disorder.

**Table 1. Clinical and demographic characteristics of infants with invasive GBS infection, stratified by EOD, LOD, and ST17 status (n=131)**

| **Characteristic** | EOD | |  | | LOD |  |  | ST17 |  |
| --- | --- | --- | --- | --- | --- | --- | --- | --- | --- |
|  | (n=37) | | NA | | (n=94) | NA | *P*-value | (n=74) | NA |
| Age at onset days, median (IQR) | 1 (1-1) | | 0 | | 24.5 (16-45.75) | 0 | - | 19.5(10.25-31) | 0 |
| Male/female ratio | 23:14 (1.64) | | 0 | | 42:52 (0.81) | 0 | >0.05 | 36:38 (0.95) | 0 |
| Birthweight (g), median (IQR) | 3060 (2780-3445) | | 2 | | 3100 (2712.5-3475) | 20 | - | 3020 (2735-3470) | 15 |
| Low birthweight (<2500 g), n (%) | 6 (17) | | 0 | | 12 (16） | 0 | >0.05 | 9 (15) | 0 |
| Length of hospital stay, days, Median (IQR) | 17 (13-24) | | 8 | | 32.5 (16.75-52) | 30 | - | 27 (16-43) | 21 |
| Mortality^a^ n (%) | 3 (9) | | 2 | | 2 (3) | 23 | >0.05 | 3 (5) | 15 |
| Preterm birth (<37 weeks), n (%) | 6 (17) | | 0 | | 13 (19) | 0 | >0.05 | 11 (19) | 0 |
| Premature rupture of membranes, n (%) | 12 (34) | 2 | | 6 (8) | | 23 | 0.002 | 7 (12) | 16 |
| Delivery mode (n), Vaginal/C-section ratio | 21:12 (1.75) | 4 | | 48:23 (2.09) | | 23 | >0.05 | 35:20 (1.75) | 19 |
| Pregnancy type, n (%), first-time | 15 (43) | 2 | | 27 (38) | | 23 | >0.05 | 22 (38) | 16 |
| GBS antenatal screening, n (%) | 8 (23) | 2 | | 6 (8) | | 23 | 0.041 | 7 (12) | 16 |
| Positive GBS antenatal screening, n (%) | 4 (50) | 29 | | 2 (33) | | 88 | >0.05 | 3 (43) | 66 |
| Maternal prenatal fever, n (%) | 3 (9) | 2 | | 2 (3) | | 23 | >0.05 | 3 (5) | 15 |
| History of Adverse pregnancy outcomes, n (%) | 6 (17) | 2 | | 5 (7) | | 23 | >0.05 | 5 (8) | 15 |
|  |  |  | |  | |  |  |  |  |

**Abbreviations**: EOD, early-onset disease (0－6 days); LOD, late-onset disease (7－89 days); IQR, interquartile range; ST, sequence type. NA, not available.

**Note**: *P values* were calculated by chi-square or Fisher’s exact test. Mortality^a^ was significantly associated with low birth weight (Fisher’s exact test, *P*=0.02). Adverse pregnancy history was defined as fetal demise or spontaneous abortion.

**

**

**Figure 1**. **Clinical and molecular distribution of invasive GBS infections in infants**

(A) Distribution of infants across hospital departments. All EOD infants and 58.5% (n=55) of LOD infants were admitted to the intensive care unit (PICU/ICU) or newborn department (including NICU). Remaining LOD patients were from the observation unit (n=17), emergency department (n=14), cranial surgery (n=5), infectious diseases department (n=2), and orthopedics department (n=1). (B) Serotype distribution patterns between EOD and LOD groups. **Abbreviations**: EOD, early-onset disease (0－6 days); LOD, late-onset disease (7－89 days); PICU, pediatric intensive care unit; ICU, intensive care unit; NICU, neonatal intensive care unit.

**3.2 Antimicrobial Susceptibility Profiles**

All 131 isolates were fully susceptible to penicillin, ampicillin, linezolid, quinupristin/dalfopristin, and vancomycin. Tetracycline (94.1%, 95/101), erythromycin (87.8%, 79/90), and clindamycin (80.4%, 82/102) exhibited high resistance rates, each exceeding 65% annually throughout the study period. Quinolone resistance fluctuated over time: low-level resistance to levofloxacin and moxifloxacin (0－25%) was observed from 2014 to 2018 but subsequently declined (**Figure 2**). Denominators for annual resistance rates varied slightly due to missing susceptibility data for some isolates in early years and differences in test panels used across periods. Resistance to ciprofloxacin, levofloxacin, and moxifloxacin was significantly associated with the ST19 lineage (*P*<0.01; **Table S2**). Detailed annual antimicrobial susceptibility profiles are provided in **Table S1**.





**Figure 2.** **Temporal trends in antimicrobial resistance to seven antimicrobial agents among invasive GBS isolates, 2014－2022.**

**Abbreviations**: CIP, ciprofloxacin; LEV, levofloxacin; MXF, moxifloxacin; CLI, clindamycin; ERY, erythromycin; TET, tetracycline; NIT, nitrofurantoin.

**3.3 Capsular Serotyping**

Four capsular serotypes (Ia, Ib, III, and V) were identified among the 131 isolates. In EOD cases, predominant serotypes were III (48.6%, 18/37) and Ia (32.4%, 12/37), followed by Ib (13.5%, 5/37) and V (5.4%, 2/37). In LOD cases, serotype III was predominant (80.9%, 76/94), followed by Ib (10.6%, 10/94) and Ia (8.5%, 8/94) (**Figure 1B**). Serotype III was associated with LOD (80.9%, 76/94; *P*<0.01), while Serotype Ia was significantly associated with EOD (60.0%, 12/20; *P*=0.01). Meningitis cases were mainly caused by serotype III (74.3%, 26/35) and serotype Ib (22.9%, 8/35). A significant association was also observed between serotype Ia and respiratory distress (61.5%, 8/13, *P*＜0.001) (**Figure 3B, Table S2)**.


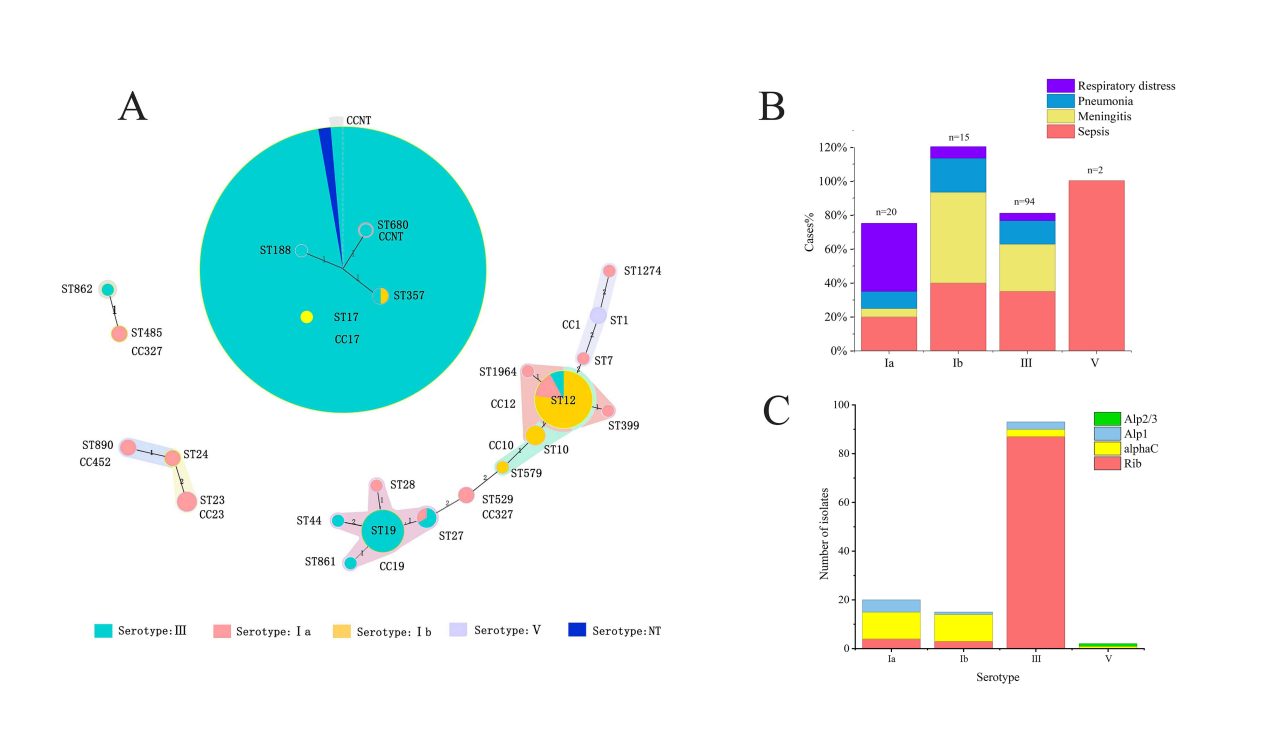


**Figure 3.** **Minimum spanning tree, infection type, and Alp gene distribution of GBS isolates**

1. Minimum spanning tree of serotypes, sequence types (STs), and clonal complexes (CCs). Circles represent STs; the size of each circle indicates the number of isolates. Colors denote serotypes; shading indicates CC assignment. Digits indicate goeBURST distance scores. (B) Serotype-specific distribution of clinical infection types. (C) Serotype-specific distribution patterns of Alp family virulence genes.

**Abbreviations**: GBS, Group B *Streptococcus*; ST, sequence type; CC, clonal complex.

**3.4 MLST and Clonal Complexes**

A total of 23 distinct STs were identified, with ST17 being the most prevalent (56.5%, 74/131), followed by ST12 (9.9%, 13/131) and ST19 (6.9%, 9/131) (**Table 2**). All STs were clustered into eight CCs, with CC17 (58.0%, 76/131), CC19 (11.5%, 15/131), and CC12 (7.6%, 10/131) being the most common (**Figure S1**). A minimum spanning tree illustrates relationships among serotype, STs, and CCs (**Figure 3A**). The III/ST17 combination accounted for 56.5% (74/131) of all isolates and was associated with LOD (*P*<0.005; **Table S2**). Other notable genotype-serotype associations include III/CC19 (9.9%, 13/131), Ib/ST12 (7.6%, 10/131), and Ib/CC10 (6.9%, 9/131).

**Table 2. Distribution of serotype and sequence types (STs) among all invasive GBS isolates and meningitis cases (n=131)**.

| **Serotype** | **STs in meningitis isolates (n, %)** | **STs in all isolates (n, %)** |
| --- | --- | --- |
| III (n=94) | ST17(22, 16.79%), ST19(3, 2.29%), ST188(1, 0.76%) | ST17(74, 56.49%), ST19(9, 6.87%), ST27(2, 1.53%), ST12(1, 0.76%), ST44(1, 0.76%), ST188(1, 0.76%), ST357(1, 0.76%), ST680(1, 0.76%), ST861(1, 0.76%), ST862(1, 0.76%) |
| Ia (n=20) | ST12(1, 0.76%) | ST23(3, 2.29%), ST12(2, 1.53%), ST24(2, 1.53%), ST485(2, 1.53%), ST529(2, 1.53%), ST890(2, 1.53%), ST27(1, 0.76%), ST28(1, 0.76%), ST399(1, 0.76%), ST7(1, 0.76%), ST1274(1, 0.76%), ST1964(1, 0.76%) |
| Ib (n=15) | ST12(4, 3.05%), ST10(2, 1.53%), ST357(1, 0.76%), ST579(1, 0.76%) | ST12(10, 7.63%), ST10(3, 2.29%), ST357(1, 0.76%), ST579(1, 0.76%) |
| V (n=2) | - | ST1(2, 1.53%) |

**Abbreviations**: GBS, group B *streptococcus*; ST, sequence type. Percentages are based on the total number of isolates.

**3.5 Temporal Distribution and Logistic Regression Analysis (2013-2017 vs. 2018-2022)**

Comparisons between the two study periods (2013－2017, n=75; 2018－2022, n=56) revealed no statistically significant differences in disease onset type or sex distribution (**Table 3**). Using serotype III as the reference, serotype Ia was more prevalent in the 2018-2022 period (32.1% vs. 2.7%; OR=13.327, 95% CI: 2.753-64.518, *P*=0.001). Serotypes Ib and V were detected exclusively in 2013-2017. Annual trends for serotypes and dominant STs are presented in **Figure 4.**

**Table 3. Comparison of disease onset, sex, and serotypes distribution during the period of 2013-2017 and 2018-2022.**

| **Characteristic** | **2013－2017（n=75）**  **n (%)** | **2018－2022（n=56）**  **n (%)** | ***P*-value** | **OR**  **(95% CI)** |
| --- | --- | --- | --- | --- |
| Disease onset |  |  |  |  |
| EOD | 19 (25.3) | 18 (33.3) | 0.392 | - |
| LOD | 56 (74.7) | 38 (66.7) |  |  |
| Sex |  |  |  |  |
| Male | 37 (49.3) | 28 (50.0) | 0.940 | - |
| Female | 38 (50.7) | 28 (50.0) |  |  |
| Serotype |  |  |  |  |
| Ia | 2 (2.7) | 18 (32.1) | 0.001 | 13.327 (2.753-64.518) |
| Ib | 15 (20.0) | 0 (0) | 0.998* | - |
| V | 2 (2.7) | 0 (0) | 0.998* |  |
| III | 56 (74.6) | 38 (67.9) | Ref. |  |

**Abbreviations**: EOD, early-onset disease; LOD, late-onset disease; OR, odds ratio; CI, confidence interval; Ref., reference.

**Note**: *Due to complete separation, Fisher’s exact test was used for serotypes Ib and V.



**Figure 4.** **Annual** **distribution of capsular serotypes and major sequence types among invasive GBS isolates, 2013－2022.**

1. Annual distribution of capsular serotypes. (B) Annual distribution of predominant sequence types (ST17, ST12, ST19, and others)

**Abbreviations**: GBS, group B *streptococcus*; ST, sequence type.

**3.6 Virulence Gene Profiles**

All 131 isolates were screened for the presence of *cylE*, *hylB*, Alp family genes, and pilus island genes (**Figure S1**). Among Alp family genes, *rib* was the most prevalent (71.8%, 94/131), followed by *alphaC* (19.8%, 26/131), *alp1* (6.9%, 9/131), and *alp2/3* (0.8%, 1/131); *alp4* was not detected. Three isolates carried no Alp family genes, and two isolates harbored two Alp genes simultaneously. The *rib* gene was significantly associated with the III/ST17 clone (74.5%, 70/94; *P*<0.001, **Table.S2**). The *alphaC* was mainly detected in serotype Ia (42.3%, 11/26) and Ib (42.3%, 11/26) isolates, and all serotype Ib/CC10 isolates (n=9) exclusively carried *alphaC* (**Figure 3C, S1**).

All isolates harbored at least one pilus island: *PI-2a* (32.1%, 42/131) or *PI-2b* (67.9%, 89/131). *PI-1* was detected in 49.6% (n=65) of isolates (**Table 4**). Pilus islands’ distribution varied significantly across serotypes and was strongly associated with specific CCs. Specifically, III/CC17 strains exclusively carried *PI-1*+*PI-2b* or *PI-2b* alone, whereas 76.9% (10/13) of III/CC19 strains carried *PI-1*+*PI-2a*. The *PI-1*+*PI-2a* or *PI-2a* genotype was present in 80% of serotype Ia (16/20) and Ib (12/15) isolates, as well as both serotype V isolates (n=2). The most prevalent virulence gene combination was *PI-2b-cylE-hylB-rib* (35.1%, 46/131) **(Figure S1)**.

**Table 4.** **Distribution of Pilus islands (Pls)stratified by capsular serotype (n=131)** .

| **Serotypes** | | **Pilus Island (n, %)** | | | | | **Total (n)** | |  |
| --- | --- | --- | --- | --- | --- | --- | --- | --- | --- |
|  |  | **PI-2a** | **PI-2b** | | **PI-1+PI-2a** | **PI-1+PI-2b** |  |  |  |
|  | Ia | 7, 5.3 | | 3, 2.3 | 9, 6.9 | 1, 0.8 | 20, 15.3 |  |  |
|  | Ib | 3, 2.3 | | 1, 0.8 | 9, 6.9 | 2, 1.5 | 15, 11.5 |  |  |
|  | III | 1, 0.8 | | 51, 38.9 | 11, 8.4 | 31, 23.7 | 94, 71.8 |  |  |
|  | V | 0 | | 0 | 2, 1.5 | 0 | 2, 1.5 |  |  |
| Total (n, %) | | 11, 8.4 | 55, 42 | | 31, 23.7 | 34, 26 | 131 | |  |
| *P** | | <0.001 | <0.001 | | <0.001 | <0.001 | <0.001 | |  |

**Abbreviations:** PI, pilus island. Data are presented as number and percentage (%). *P** values were calculated by Fisher’s exact test.

1. **Discussion**

GBS is an important cause of sepsis and meningitis in newborns worldwide, with preterm infants at particularly high risk. While IAP effectively reduces the incidence of EOD, it is not fully protective, and rare cases of EOD still occur even among infants born to mothers who received guideline-recommended IAP^21^. Beyond antibiotic resistance, the biofilm-forming capacity of GBS has been reported to be associated with IAP failure and persistent maternal colonization^22^.

Invasive GBS infections continue to pose a substantial clinical burden for infants at the study center, with a median of 12.5 cases annually (IQR: 6.5–19.25) during 2013–2022. Consistent with data from Singapore^23^, we observed a higher incidence of LOD than EOD (**Table 1**), a pattern that differs from that reported in South Africa^24^, likely reflecting regional variations in IAP implementation and clinical practice^25^. The median age at EOD onset was 1 day (IQR: 1-1), consistent with vertical transmission from colonized mothers and known obstetric risk factors, such as premature rupture of membranes and maternal fever^26^. In contrast, LOD typically has a subacute presentation, with potential transmission routes including postnatal maternal contact (e.g., breast milk), horizontal transmission from household contacts, community acquisition, or nosocomial spread^27^.

The *Chinese Experts’ Consensus on Prevention of Perinatal GBS Disease* was issued in August 2021^28^. In this study, approximately 86.8% of pregnant women had no GBS screening or no documented results, and two-thirds of available screening records were obtained after August 2021. These findings highlight the necessity to promote standardized prenatal GBS screening and consistent IAP implementation across healthcare facilities in China.

The distribution of GBS serotypes and STs varies geographically^29-31^. In this study, the III/ST17 clone (56.5%) was the predominant lineage among infantile GBS isolates, a prevalence higher than the Chinese national average of 40.6%^28^. We confirmed a significant association between LOD and III/ST17 (*P*<0.005), consistent with European studies linking CC17 to hypervirulence, neonatal meningitis, enhanced blood-brain barrier penetration, and immune evasion^32-34^. A notable epidemiological shift was observed between 2013-2017 and 2018-2022; the prevalence of serotype Ia increased from 2.7% to 33.3% (*P*＜0.05, OR=13.327), while serotype Ib decreased from 20.0% to 0% (**Table 3**). This shift may be related to clonal expansion of fitter serotype Ia lineages, potentially driven by capsular switching^35^. We also identified a significant association between serotype Ia and respiratory distress/failure, consistent with previous studies in southern China linking serotype Ia to pneumonia^17^, suggesting the clinical value of molecular serotyping for early risk stratification and targeted management of invasive GBS disease.

All GBS isolates in this study remained susceptible to penicillin, ampicillin, and vancomycin, while resistance rates to erythromycin, clindamycin, and tetracycline each exceeded 80%. This susceptibility profile aligns with recent epidemiological data from mainland China, confirming penicillin as the first-line agent and reporting a pooled erythromycin resistance rate of 65.4%, with geographically variable clindamycin resistance (exceeding 70% in northeast China)^36^. High macrolide and lincosamide resistance is a well-documented global trend^37^, driven mainly by *erm* genes (ribosomal target modification) and *mefA*/*E* genes (efflux-mediated resistance)^36,37^. Tetracycline resistance (>80% nationally) is mainly attributed to *tetM* and *tetO*^36,37^. Multidrug resistance was detected in 66.7% (10/15) of fluoroquinolone-resistant isolates, particularly in III/ST19 (50%, 5/10); 80% (4/5) of these isolates were coresistant to fluoroquinolones and lincosamides, macrolides, or tetracyclines. The underlying mechanisms may involve fluoroquinolone target gene mutations and resistance clusters such as *lnu(B)*, disseminated via integrative conjugative elements or transposons^19,38^.

Vertical transmission is a primary route and an important reservoir for LOD^39,40^. The *hylB* (hyaluronidase) and *cylE* (hemolysin) genes are key virulence determinants that facilitate GBS across of the placental barrier and establishment of neonatal infection^41,42^. The *cylE* gene, located within the *cyl* operon, was originally thought to encode the β-hemolysin/cytolysin toxin but is now recognized as an acyl‑CoA acyltransferase that catalyzes the biosynthesis of the hemolytic glycolipid ornithine rhamnolipid; deletion of *cylE* abrogates toxin production and results in a non‑hemolytic phenotype^43,44^. The *hylB* gene encodes a secreted hyaluronate lyase that degrades hyaluronic acid in the extracellular matrix, thereby disrupting tissue integrity and facilitating bacterial dissemination implicated in ascending uterine infection and adverse pregnancy outcomes, including preterm birth, stillbirth, and fetal injury^41^.Both the *cylE* and *hylB* genes were detected in all isolates (100%), consistent with reports from Fuzhou, China^45^, and the Argentine Pampa region^46^. Given their universal presence and important role in GBS pathogenesis, inhibitors targeting *cylE* or *hylB* may represent potential adjunct strategies to conventional IAP for reducing EOD incidence.

Despite the implementation of IAP, EOD cases still occur worldwide^47^, potentially attributed to biofilm formation in GBS-colonized mothers. Pilus islands (*PI-1*, *PI-2a*, and *PI-2b*) are essential for biofilm formation and GBS virulence^48^. Consistent with previous Chinese reports^49,50^, *PI-2b* was universally detected in III/ST17 isolates in our study and has been linked to enhanced macrophage internalization and intracellular survival^51^. *PI-2a*, associated with biofilm formation in chronic infections and antibiotic resistance^52^, was carried by >75% of non-III/ST17 isolates (**Table 4 and Figure S1**). The III/ST17 lineage exhibit biofilm formation independent of pilus genes, relying instead on capsular polysaccharides, biofilm regulatory proteins, and CsrR-mediated regulation of the adhesin *BapB*^53,54^. One EOD case occurred despite maternal IAP; the causative isolate was the serotype III/CC19 strain fully susceptible to penicillin and clindamycin and harboring *PI-2a* and *PI-1*, indicating that IAP failure may be associated with biofilm formation in this case.

Vaccine development is important for LOD prevention, although no licensed GBS vaccine is currently available. Surface protein genes (Alp family) are important targets for vaccine design. In this study, *rib* was the most prevalent Alp family gene (71.8%, 94/131) and strongly associated with serotype III, suggesting a potential neurotropic role^55^. A hexavalent capsular polysaccharide-conjugate vaccine targeting serotypes Ia, Ib, and Ⅱ-V would cover all isolates in this study^56^. Alp family protein-based vaccines (e.g., *alphaC*, *rib*, *alp1*, and *alp2/3*) could offer broader protection^57^ and would cover 97.7% of our isolates. Additional strategies, including predictive models integrating clinical and molecular data for highest-risk neonates and biofilm-inhibiting agents, may further reduce the clinical burden of GBS infections, especially LOD.

With a median of 12.5 annual cases at our center, LOD complicated by meningitis and neurological sequelae (including epilepsy, speech delay, and autism spectrum disorder in three of our patients) constitutes a substantial clinical burden. Therefore, standardized long-term multidisciplinary neurodevelopmental follow-up and timely rehabilitation are important for infants surviving GBS meningitis. Early identification of subtle developmental disorders, prompt targeted rehabilitation, and individualized early intervention are necessary to minimize long-term neurological sequelae, maximize developmental potential, and improve long-term quality of life. These findings further support the importance of effective prevention, optimized clinical management, and refined risk stratification for neonatal invasive GBS disease.

Several limitations should be addressed in this study. First, this was a single-center retrospective study, which may restrict the external validity and generalizability of our conclusions. Second, the limited follow-up duration resulted in insufficient data on long-term neurodevelopmental outcomes of survivors. Third, the exclusion of mild and outpatient GBS cases may introduce selection bias. Finally, although multivariate analysis was performed, unmeasured or residual confounding factors could not be entirely excluded.

**Conclusion**

Invasive GBS infections in infants aged ≤90 days in Guangzhou were associated with the hypervirulent III/ST17 clone, and serotype Ia was associated with respiratory distress in EOD cases. No substantial shifts in antimicrobial resistance patterns were observed over the 10-year study period, indicating that penicillin and ampicillin were still the first-line agents for GBS prophylaxis and treatment. Our findings highlight the necessity to strengthen prenatal GBS screening and improve IAP implementation in China. Given that cases of IAP failure may be related to biofilm, vaccine development and nonvaccine approaches (e.g., anti-biofilm agents or monoclonal antibodies targeting key virulence factors) should be considered to help reduce the burden of infantile invasive GBS disease.

## **Ethics** Approval and Consent to Participate

This study was conducted in accordance with the Declaration of Helsinki and approved by the Ethics Committee of Guangzhou Medical University Affiliated Women and Children’s Medical Center, Guangzhou, China (approval no. 2025120A01). Individual consent for participation was waived by the IRB of Guangzhou Medical University Affiliated Women and Children’s Medical Center due to the retrospective nature of the study. All clinical data were anonymized and de-identified.

**Author Contributions**

Lian-fen Huang conceived and designed the experiments. Xiao-lan Chen, Wen-shan Chen, Li-Jia Wei, Kan-kan Gao, and Lian-fen Huang performed the experiments. Lian-fen Huang, Hua-min Zhong, Yong-qiang Xie, Kan-kan Gao, Bing-shao Liang, Yun-feng Liu, Fei Gao, Yan Long collected/provided the clinical samples and data. Xiao-lan Chen, Lian-fen Huang, Wen-shan Chen, Li-Jia Wei drafted and revised the manuscript with input from all authors. All authors read and approved the final manuscript.

**Funding**

This work was supported by grants from the Natural Science Foundation of Guangdong Province, China (2026A1515012832); Guangzhou Science, Technology and Innovation Commission and Guangzhou Children and Women Medical Center (2023A03J0927); and Guangdong Provincial Clinical Research Center for Laboratory Medicine(2023B110008).

**Conflict Of Interest**

The authors declare that they have no conflicts of interest.

**Consent for publication**

Not applicable, as the manuscript contains no individual person’s data (e.g., images, videos, or personal details) that would require consent for publication.

**Data Availability Statement**

All data generated and analyzed during this study are included in this published article and its supplementary materials. Individual-level clinical datasets are not publicly available owing to patient privacy protections and institutional ethical regulations. De-identified individual-level data may be obtained from the corresponding author upon reasonable formal request, subject to prior official approval by the Institutional Review Board of Guangzhou Medical University Affiliated Women and Children’s Medical Center.

**References**

1. Nanduri SA, Petit S, Smelser C, et al. Epidemiology of invasive early-onset and late-onset group B streptococcal disease in the United States, 2006 to 2015: multistate laboratory and population-based surveillance. JAMA Pediatr, 2019. 173(3):224-233.doi: 10.1001/jamapediatrics.2018.4826.
2. Ji W, Wang L, Zhu F, et al. Clinical and molecular epidemiology of invasive group B streptococcal disease among infants, China. Emerg Infect Dis, 2019, 25(11):2021-2030. doi: 10.3201/eid2511.190569.
3. Alotaibi NM, Alshahrani S, Alshahrani A, et al. Clinical characteristics and treatment strategies for group B *streptococcus* (GBS) infection in pediatrics: a systematic review. Medicina (Kaunas), 2023, 59(7):1218. doi: 10.3390/medicina59071218.
4. Liu Y, Liu J. Group B *streptococcus*: virulence factors and pathogenic mechanism. Microorganisms, 2022, 10(12):2407. doi: 10.3390/microorganisms10122407.
5. Alvim D, Bellei N, Lincopan N, et al. Biofilm production and distribution of pilus variants among *Streptococcus agalactiae* isolated from human and animal sources. Biofouling, 2019, 35(8):938-944. doi: 10.1080/08927014.2019.1653257.
6. Pawlowski A, Krachler A, Xia Y, et al. A group B *streptococcus* alpha-like protein subunit vaccine induces functionally active antibodies in humans targeting homotypic and heterotypic strains. Cell Rep Med, 2022, 3(2):100511. doi: 10.1016/j.xcrm.2022.100511.
7. Randis TM, Gelber SE, Aguilar JL, et al. Group B *streptococcus* β-hemolysin/cytolysin breaches maternal-fetal barriers to cause preterm birth and intrauterine fetal demise in vivo. J Infect Dis, 2014, 210(2):265-273. doi: 10.1093/infdis/jiu130.
8. Vornhagen J, Quach D, Gendrin C, et al. Bacterial hyaluronidase promotes ascending GBS infection and preterm birth. mBio, 2016, 7(3):e00741-16. doi: 10.1128/mBio.00741-16.
9. Kong F, Gilbert GL, Gordon DL, et al. Serotype identification of group B *streptococci* by PCR and sequencing. J Clin Microbiol, 2002, 40(1):216-226. doi: 10.1128/JCM.40.1.216-226.2002.
10. Poyart C, Tazi A, Régnault B, et al. Multiplex PCR assay for rapid and accurate capsular typing of group B *streptococci*. J Clin Microbiol, 2007, 45(6):1985-1988. doi: 10.1128/JCM.00244-07.
11. Imperi M, Pataracchia M, Alfarone G, et al. A multiplex PCR assay for the direct identification of the capsular type (Ia to IX) of *Streptococcus agalactiae*. J Microbiol Methods, 2010, 80(2):212-214. doi: 10.1016/j.mimet.2009.12.002.
12. Jones N, Bohnsack JF, Takahashi S, et al. Multilocus sequence typing system for group B *streptococcus*. J Clin Microbiol, 2003, 41(6):2530-2536. doi: 10.1128/JCM.41.6.2530-2536.2003.
13. Gori A, Gutiérrez-Cabrera AE, Bottova I, et al. Pan-GWAS of *Streptococcus agalactiae* highlights lineage-specific genes associated with virulence and niche adaptation. mBio, 2020, 11(3):e00629-20. doi: 10.1128/mBio.00629-20.
14. Hsu JF, Chen YH, Chen SY, et al. The clinical and genetic characteristics of *Streptococcus agalactiae* meningitis in neonates. Int J Mol Sci, 2023, 24(20):15128. doi: 10.3390/ijms242015128.
15. Li J, Chen X, Zhong H, et al. Molecular characteristics of group B *streptococcus* isolates from infants in southern mainland China. BMC Infect Dis, 2019, 19(1):1068. doi: 10.1186/s12879-019-4616-2.
16. Wang X, Li Y, Chen Y, et al. Phenotypic and molecular characterization of *Streptococcus agalactiae* colonized in Chinese pregnant women: predominance of ST19/III and ST17/III. Res Microbiol, 2018, 169(2):101-107. doi: 10.1016/j.resmic.2017.10.004.
17. Zhu Y, Li J, Chen X, et al. Etiological serotype and genotype distributions and clinical characteristics of group B *streptococcus*-inducing invasive disease among infants in South China. BMC Pediatr, 2020, 20(1):146. doi: 10.1186/s12887-020-1969-9.
18. Arias B, Ponce V, Papalia M, et al. Fluoroquinolone-resistant *Streptococcus agalactiae* invasive isolates recovered in Argentina. Microb Drug Resist, 2019, 25(5):739-743. doi: 10.1089/mdr.2018.0232.
19. Gao K, Chen X, Zhong H, et al. Predominance of III/ST19 and Ib/ST10 lineages with high multidrug resistance in fluoroquinolone-resistant group B *streptococci* isolates in which a new integrative and conjugative element was identified. Front Microbiol, 2021, 12:642455. doi: 10.3389/fmicb.2021.642455.
20. Zhang L, Gao H, Wang Y, et al. Emergence of invasive serotype Ib sequence type 10 group B *streptococcus* disease in Chinese infants is driven by a tetracycline-sensitive clone. Front Cell Infect Microbiol, 2021, 11:642455. doi: 10.3389/fcimb.2021.642455.
21. Blaschke AJ, Ferrieri P, Huie ME, et al. Clindamycin-resistant group B *streptococcus* and failure of intrapartum prophylaxis to prevent early-onset disease. J Pediatr, 2010, 156(3):501-503. doi: 10.1016/j.jpeds.2009.10.012.
22. Pell ME, Bonsch C, Hays AP, et al. Genomic adaptation in group B *streptococcus* following intrapartum antibiotic prophylaxis and childbirth. bioRxiv, 2025. doi: 10.1101/2025.01.07.243021.
23. Kam KQ, Tan EL, Ang B, et al. Serotype distribution and incidence of invasive early onset and late onset group B streptococcal disease amongst infants in Singapore. BMC Infect Dis, 2021, 21(1):1221. doi: 10.1186/s12879-021-05934-8.
24. Dangor Z, Cutland CL, Izu A, et al. Temporal changes in invasive group B streptococcal serotypes: implications for vaccine development. PLoS One, 2016, 11(12):e0169101. doi: 10.1371/journal.pone.0169101.
25. Verani JR, McGee L, Schrag SJ. Prevention of perinatal group B streptococcal disease—revised guidelines from CDC, 2010. MMWR Recomm Rep, 2010, 59(RR-10):1-36. doi: 10.15585/mmwr.rr5910a1.
26. Oddie SJ, Embleton ND. Risk factors for early onset neonatal group B streptococcal sepsis: case-control study. BMJ, 2002, 325(7359):308. doi: 10.1136/bmj.325.7359.308.
27. Hon KL, Leung AKC, Lam RKK. Late-onset group B streptococcal disease is a rare but devastating disease. Hong Kong Med J, 2021, 27(3):229-230. doi: 10.12809/hkmj.20200996.
28. Chinese Medical Association Obstetrics and Gynecology Branch. Chinese experts consensus on prevention of perinatal group B streptococcal disease. Chin J Perinat Med, 2021, 24(8):561-566. doi: 10.3760/cma.j.cn113903-20210616-00531.
29. Tsai MH, Chen YH, Huang JY, et al. Molecular characteristics and antimicrobial resistance of group B *streptococcus* strains causing invasive disease in neonates and adults. Front Microbiol, 2019, 10:264. doi: 10.3389/fmicb.2019.00264.
30. Meehan M, Cunney RJ, Cafferkey MT. Genomic epidemiology of group B *streptococci* spanning 10 years in an Irish maternity hospital, 2008-2017. J Infect, 2021, 83(1):37-45. doi: 10.1016/j.jinf.2021.03.005.
31. Alzayer M, Alghamdi MA, Althobiti M, et al. Genomic insights into the diversity, virulence, and antimicrobial resistance of group B *streptococcus* clinical isolates from Saudi Arabia. Front Cell Infect Microbiol, 2024, 14:1377993. doi: 10.3389/fcimb.2024.1377993.
32. Romain AS, Dmytruk N, Bidet P, et al. Clinical and laboratory features of group B *streptococcus* meningitis in infants and newborns: study of 848 cases in France, 2001-2014. Clin Infect Dis, 2018, 66(6):857-864. doi: 10.1093/cid/cix943.
33. Tazi A, Bidet P, Dmytruk N, et al. Risk factors for infant colonization by hypervirulent CC17 group B *streptococcus*: toward the understanding of late-onset disease. Clin Infect Dis, 2019, 69(10):1740-1748. doi: 10.1093/cid/ciz155.
34. Deshayes de Cambronne R, Boudet F, Bidet P, et al. CC17 group B *streptococcus* exploits integrins for neonatal meningitis development. J Clin Invest, 2021, 131(5):e145688. doi: 10.1172/JCI145688.
35. Khan UB, Jones CH, Harrison OB, et al. Identifying large-scale recombination and capsular switching events in *Streptococcus agalactiae* strains causing disease in adults in the UK between 2014 and 2015. Microb Genom, 2022, 8(3):mgen000836. doi: 10.1099/mgen.0.000836.
36. Guo D, Wang L, Ye K, et al. Neonatal colonization of group B *streptococcus* in China: prevalence, antimicrobial resistance, serotypes, and molecular characterization. Am J Infect Control, 2018, 46(3):e19-e24. doi: 10.1016/j.ajic.2017.10.014.
37. Hayes K, O’Halloran F, Cotter L. A review of antibiotic resistance in group B *streptococcus*: the story so far. Crit Rev Microbiol, 2020, 46(3):253-269. doi: 10.1080/1040841X.2020.1744344.
38. Zhou K, Chen X, Zhong H, et al. New genetic context of lnu(B) composed of two multi-resistance gene clusters in clinical *Streptococcus agalactiae* ST-19 strains. Antimicrob Resist Infect Control, 2019, 8:117. doi: 10.1186/s13756-019-0569-7.
39. Tavares T, Pinho L, Andrade EB. Group B streptococcal neonatal meningitis. Clin Microbiol Rev, 2022, 35(2):e00079-21. doi: 10.1128/CMR.00079-21.
40. Berardi A, Rossi C, Mammini A, et al. Understanding factors in group B *streptococcus* late-onset disease. Infect Drug Resist, 2021, 14:3207-3218. doi: 10.2147/IDR.S337132.
41. Coleman K, Vornhagen J, Gendrin C, et al. Hyaluronidase impairs neutrophil function and promotes group B *streptococcus* invasion and preterm labor in nonhuman primates. mBio, 2021, 12(1):e03710-20. doi: 10.1128/mBio.03710-20.
42. Yang R, Wang Y, Li X, et al. Blood-brain barrier integrity damage in bacterial meningitis: the underlying link, mechanisms, and therapeutic targets. Int J Mol Sci, 2023, 24(3):2407. doi: 10.3390/ijms24032407.
43. Liu GY, Doran KS, Lawrence T, et al. Sword and shield: linked group B streptococcal beta-hemolysin/cytolysin and carotenoid pigment function to subvert host phagocyte defense. Proc Natl Acad Sci U S A, 2004, 101(40):14491-14496. doi: 10.1073/pnas.0402842101.
44. Rosa-Fraile M, Dramsi S, Spellerberg B. Group B streptococcal haemolysin and pigment, a tale of twins. FEMS Microbiol Rev, 2014, 38(5):932-946. doi: 10.1111/1574-6976.12066.
45. Liang B, Chen X, Zhong H, et al. Molecular epidemiology of group B *streptococcus* isolates from pregnant women with premature rupture of membranes in Fuzhou, China. Infect Drug Resist, 2023, 16:269-278. doi: 10.2147/IDR.S396231.
46. Hernandez LB, Famiglietti A, Vay C, et al. Virulence profiles and antimicrobial resistance of *Streptococcus agalactiae* infective and colonizing strains from Argentina. Curr Microbiol, 2022, 79(12):392. doi: 10.1007/s00284-022-03013-2.
47. Le Doare K, Seedat A, Jackson S, et al. Intrapartum antibiotic chemoprophylaxis policies for the prevention of group B streptococcal disease worldwide: systematic review. Clin Infect Dis, 2017, 65(Suppl 2):S143-S151. doi: 10.1093/cid/cix234.
48. Rosini R, Rinaudo CD, Soriani M, et al. Identification of novel genomic islands coding for antigenic pilus-like structures in *Streptococcus agalactiae*. Mol Microbiol, 2006, 61(1):126-141. doi: 10.1111/j.1365-2958.2006.05210.x.
49. Lu B, Chen X, Zhong H, et al. Microbiological and clinical characteristics of group B *streptococcus* isolates causing materno-neonatal infections: high prevalence of CC17/PI-1 and PI-2b sublineage in neonatal infections. J Med Microbiol, 2018, 67(11):1551-1559. doi: 10.1099/jmm.0.000811.
50. Zhang L, Gao H, Wang Y, et al. Molecular characterization of pathogenic group B *streptococcus* from a tertiary hospital in Shanxi, China: high incidence of sequence type 10 strains in infants/pregnant women. J Microbiol Immunol Infect, 2021, 54(6):1094-1100. doi: 10.1016/j.jmii.2020.08.006.
51. Périchon B, Gaultier E, Dramsi S, et al. Insights into *Streptococcus agalactiae* PI-2b pilus biosynthesis and role in adherence to host cells. Microbes Infect, 2019, 21(2):99-103. doi: 10.1016/j.micinf.2018.11.003.
52. Rinaudo CD, Rosini R, Soriani M, et al. Specific involvement of pilus type 2a in biofilm formation in group B *streptococcus*. PLoS One, 2010, 5(2):e9216. doi: 10.1371/journal.pone.0009216.
53. D'Urzo N, Morniroli D, Biagini M, et al. Acidic pH strongly enhances in vitro biofilm formation by a subset of hypervirulent ST-17 *Streptococcus agalactiae* strains. Appl Environ Microbiol, 2014, 80(7):2176-2185. doi: 10.1128/AEM.03864-13.
54. Li W, Chen X, Zhong H, et al. CsrR modulates biofilm formation of ST-17 GBS through the regulation of a novel adhesion factor, BapB. Microb Pathog, 2025, 207:107873. doi: 10.1016/j.micpath.2024.107873.
55. Pawlowski A, Krachler A, Xia Y, et al. A group B *streptococcus* alpha-like protein subunit vaccine induces functionally active antibodies in humans targeting homotypic and heterotypic strains. Cell Rep Med, 2022, 3(2):100511. doi: 10.1016/j.xcrm.2022.100511.
56. Absalon J, Schmoetten M, Kacheva I, et al. Safety and immunogenicity of a novel hexavalent group B *streptococcus* conjugate vaccine in healthy, non-pregnant adults: a phase 1/2, randomised, placebo-controlled, observer-blinded, dose-escalation trial. Lancet Infect Dis, 2021, 21(2):263-274. doi: 10.1016/S1473-3099(20)30765-8.
57. Gonzalez-Miro C, Barrera C, Arias C, et al. Safety and immunogenicity of the group B *streptococcus* vaccine AlpN in a placebo-controlled double-blind phase 1 trial. iScience, 2023, 26(3):106261. doi: 10.1016/j.isci.2023.106261.
